# Efficacy of multidomain interventions to improve physical frailty, depression and cognition: data from cluster‐randomized controlled trials

**DOI:** 10.1002/jcsm.12534

**Published:** 2020-03-05

**Authors:** Liang‐Kung Chen, An‐Chun Hwang, Wei‐Ju Lee, Li‐Ning Peng, Ming‐Hsien Lin, David L. Neil, Shu‐Fang Shih, Ching‐Hui Loh, Shu‐Ti Chiou, Taiwan Health Promotion Intervention Study for Elders research group

**Affiliations:** ^1^ Center for Geriatrics and Gerontology Taipei Veterans General Hospital Taipei Taiwan; ^2^ Aging and Health Research Center National Yang Ming University Taipei Taiwan; ^3^ Department of Geriatric Medicine National Yang Ming University School of Medicine Taipei Taiwan; ^4^ Department of Family Medicine Taipei Veterans General Hospital Yuanshan Branch Yilan Taiwan; ^5^ Full Universe Integrated Marketing Taipei Taiwan; ^6^ Department of Health Management and Policy University of Michigan Ann Arbor Michigan USA; ^7^ Hualien Tzu Chi Hospital Buddhist Tzu Chi Medical Foundation Hualien Taiwan; ^8^ Institute of Public Health National Yang Ming University Taipei Taiwan; ^9^ Cheng‐Hsin General Hospital Taipei Taiwan

**Keywords:** Healthy aging, Physical frailty, Multidomain intervention, Community, Elder empowerment, Cognitive, Malnutrition, Outcome

## Abstract

**Background:**

Frailty is the pre‐eminent exigency of aging. Although frailty‐related impairments are preventable, and multidomain interventions appear more effective than unimodal ones, the optimal components remain uncertain.

**Methods:**

We devised multidomain interventions against physical and cognitive decline among prefrail/frail community‐dwelling ≥65‐year‐olds and evaluated these in complementary cluster‐randomized trials of efficacy and participant empowerment. The *Efficacy Study* compared ~3‐monthly telephone consultations vs. 16, 2 h sessions/year comprising communally partaken physical and cognitive training plus nutrition and disease education; the *Empowerment Study* compared the standard *Efficacy Study* multidomain intervention (Sessions 1–10) vs. an enhanced version redesigned to empower and motivate individual participants. Changes from baseline in physical, functional, and cognitive performance were measured after 6 and 12 months in the *Efficacy Study* and after 6 months in the *Empowerment Study*, with post‐intervention follow‐up at 9 months. Primary outcomes are as follows: Cardiovascular Health Study frailty score; gait speed; handgrip strength; and Montreal Cognitive Assessment (MoCA). Secondary outcomes are as follows: instrumental activities of daily living; metabolic equivalent of task (MET); depressed mood (Geriatric Depression Scale‐5 ≥2); and malnutrition (Mini‐Nutritional Assessment short‐form ≤11). Intervention effects were analyzed using a generalized linear mixed model.

**Results:**

*Efficacy Study* participants (*n* = 1082, 40 clusters) were 75.1 ± 6.3 years old, 68.7% women, and 64.7% prefrail/frail; analytic clusters: 19 intervention (410/549 completed) vs. 21 control (375/533 completed). *Empowerment Study* participants (*n* = 440, 14 clusters) were 75.9 ± 7.1 years old, 83.6% women, and 56.7% prefrail/frail; analytic clusters: seven intervention (209/230 completed) vs. seven control (189/210 completed). The standard and enhanced multidomain interventions both reduced frailty and significantly improved aspects of physical, functional, and cognitive performance, especially among ≥75‐year‐olds. Standard multidomain intervention decreased depression [odds ratio 0.56, 95% confidence interval (CI) 0.32, 0.99] and malnutrition (odds ratio 0.45, 95% CI 0.26, 0.78) by 12 months and improved concentration at Months 6 (0.23, 95% CI 0.04, 0.42) and 12 (0.46, 95% CI 0.22, 0.70). Participant empowerment augmented activity (4.67 MET/h, 95% CI 1.64, 7.69) and gait speed (0.06 m/s, 95% CI 0.00, 0.11) at 6 months, with sustained improvements in delayed recall (0.63, 95% CI 0.20, 1.06) and MoCA performance (1.29, 95% CI 0.54, 2.03), and less prevalent malnutrition (odds ratio 0.39, 95% CI 0.18, 0.84), 3 months after the intervention ceased.

**Conclusions:**

Pragmatic multidomain intervention can diminish physical frailty, malnutrition, and depression and enhance cognitive performance among community‐dwelling elders, especially ≥75‐year‐olds; this might supplement healthy aging policies, probably more effectively if participants are empowered.

## Introduction

Population aging is a global problem, imposing substantial and rapidly increasing health care and socio‐economic burdens.^1^, ^2^ Its most exigent manifestation is frailty, a distinct geriatric phenotype prognostic of disability, loss of independence, and earlier death, irrespective of age or morbidity status.^1^, ^3^, ^4^, ^5^ Frail individuals are less likely and slower to recover from injury or stressful life events, steepening the trajectory of physical, functional, and cognitive decline.^1^, ^4^ The prevalence of frailty among community‐dwelling ≥65‐year‐olds from predominantly Europid populations averages approximately 10–12%, within wide bounds.^6^, ^7^ Until recently, frailty appeared relatively less common in East Asians,^7^, ^8^, ^9^ but this is changing; Taiwan has the most rapidly aging populace in the world, which is expected to transition from aged (14% of the population ≥65 years old) in 2018 to super‐aged (20% ≥65 years old) in less than 10 years, with profound health policy implications.^2^, ^10^ For these reasons, ‘healthy aging’ to promote well‐being and forfend age‐related ill health has become an international priority.^10^, ^11^

The nexus of ‘phenotypic’ frailty involves loss of muscle strength and mass, impaired locomotion, diminishing physical function, and fatigue.^1^, ^4^, ^12^ Though complex, there is strong evidence that these factors are modifiable, making them salient targets for preventing or postponing the adverse consequences of frailty.^1^, ^4^, ^5^, ^13^, ^14^, ^15^ However, devising pragmatic and demonstrably effective interventions for this multifaceted condition has proven challenging.^15^, ^16^ Although numerous studies have targeted various aspects of frailty or disability in older people, particularly those relating to physical performance, the results have been mixed, besides being difficult to compare due to differing inclusion criteria, methodologies, and operational definitions of frailty.^14^, ^15^, ^16^, ^17^, ^18^, ^19^ Few studies have recruited participants based on specific frailty criteria—fewer still evaluated frailty itself as a primary outcome.^14^, ^15^, ^20^ Consequently, it remains uncertain which approaches are most likely to be effective and economically expedient,^15^, ^16^, ^18^, ^19^ although there is consensus that exercise training can prevent or delay the onset of physical frailty.^15^, ^17^, ^18^ Emerging evidence supports conjecture that multidomain interventions that address complex individual care needs might be more advantageous than those focused on specific diseases or deficits, but further research is needed to resolve current uncertainties.^1^, ^15^, ^16^, ^19^ There is a dearth of research on cognitive or psychosocial factors, despite their probable role in bolstering resilience in old age.^4^, ^15^, ^17^, ^18^, ^21^

To advance healthier aging on a global scale, interventions to prevent frailty must be pragmatic, affordable, and generalizable to different societal structures and circumstances. To this end, we developed two community‐based multidomain interventions, administered using simple resources, and evaluated their effect in preventing physical and cognitive decline among senior citizens at risk of adverse frailty‐related outcomes. We emulated contemporary trials of lifestyle interventions in older people in using a cluster‐randomized design,^22^, ^23^, ^24^, ^25^ which is expedient and facilitates robust comparative analyses in such settings. We observed significant improvements in aspects of physical, cognitive, and functional performance among elders who participated in both multidomain interventions and report new evidence that such interventions were most effective among participants who were empowered, and especially beneficial in older participants (≥75 years).

## Methods

### Design and participants

Taiwan Health Promotion Intervention Study for Elders comprised complementary prospective cluster‐randomized trials (Supporting Information, *Figure* *S1*), conducted from 2014 to 2017: one assessed the efficacy of a 12 month participatory community‐group multidomain intervention against physical and cognitive decline among prefrail/frail community‐dwelling older people (*Efficacy Study*); the other evaluated the benefit of further empowering individual participants (*Empowerment Study*). Trials designed to compare interventions that entail group activities involving participants from single communities have inherent problems with contamination and participant blinding. Therefore, we used a cluster‐randomized design, both to control for between‐group contamination and to facilitate evaluation of implementation effectiveness. Unlike conventional randomized controlled trials, which compare intervention effects on individual outcomes, cluster randomization allows powerful extrapolation of the findings to the entire community (cluster) studied.

**Figure 1:**
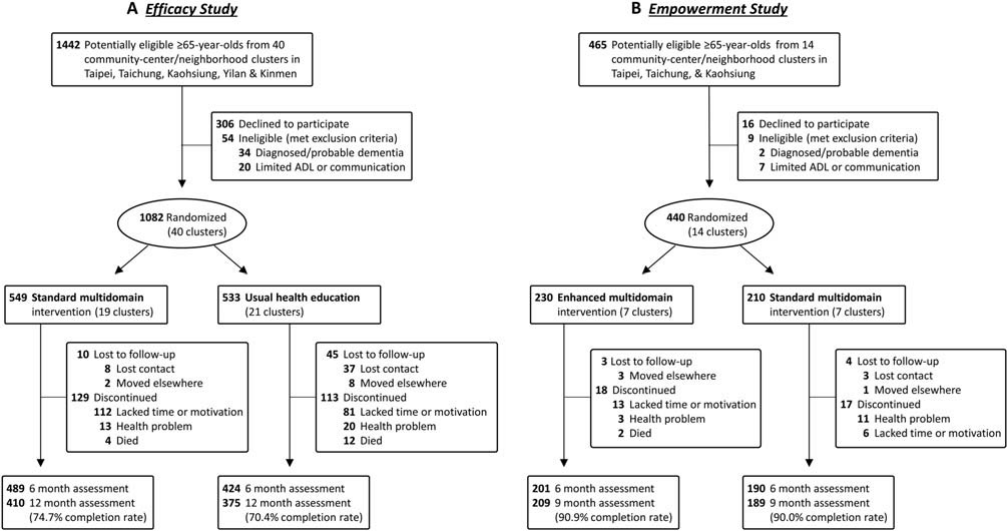
*Efficacy and Empowerment Studies*: participant selection, randomization, and disposition. ADL, activities of daily living.

The *Efficacy Study* enrolled participants from 40 clusters (community centers/neighborhoods with 500–1000 residents ≥65 years old) in five cities/counties across Taiwan: Taipei, Taichung, and Kaohsiung, Yilan, and Kinmen; the *Empowerment Study* enrolled a separate cohort of participants, who did not overlap with those in the *Efficacy Study*, from another 14 clusters in Taipei, Taichung, and Kaohsiung (*Figure* 1). From 12 February 2014 until 5 May 2016, trained staff visited community centers to tell local residents about the study and interviewed potential participants to assess their eligibility. The inclusion criteria were age ≥65 years; currently receiving Taiwan National Health Insurance services; subjective memory impairment and/or loss of ≥1 instrumental activities of daily living (IADL), and/or timed 6 m walk speed ≤1 m/s; and competence to sign informed consent personally and to comply with study procedures. Exclusion criteria were age <65 years; dementia diagnosed according to the *Diagnostic and Statistical Manual of Mental Disorders, 4th Edition* 26 or suspected by a clinician; self/caregiver‐reported total or partial dependence for ADL, or major illness with life expectancy <6 months; interviewer‐adjudicated severe hearing or visual impairment; documented major depression or anxiety, or other major illness that may jeopardize compliance (at investigators' discretion); institutionalization; or current participation in other clinical studies or research. Independent researchers not involved in assessing outcomes used a random number sequence generated by Excel 2013 (Microsoft, Redmond, WA, USA) to allocate participants in clusters, by simple direct sampling, 1:1 to intervention or control groups. Opaque sealed envelopes were used to conceal the interventions allocated from participants and assessors.

### Ethical compliance and trial registration

The Taiwan Health Promotion Intervention Study for Elders was conducted according to the ethical standards established by the 1964 Declaration of Helsinki and later amendments, and prevailing national regulations and guidelines. The Joint Institutional Review Board of Taiwan reviewed and approved the trial protocols (JIRB No. 14‐001‐A). All study participants provided written informed consent before any study‐related procedure ensued.

The Taiwan Health Promotion Intervention Study for Elders was registered retrospectively, on 17 February 2017, at ClinicalTrials.gov: NCT03056768.

### Procedures

The *Efficacy Study* compared the effect of multidomain intervention over 12 months with conventional health education, and the *Empowerment Study* compared implementing the same standard multidomain intervention as was used in the *Efficacy Study* for 6 months, vs. an enhanced version of that programme, with post‐intervention follow‐up at 9 months (Supporting Information, *Figure* *S1*).

The multidomain interventions were administered by appropriate professionals (e.g. fitness coach, physical therapist, occupational therapist, and dietician) or by trained staff. Before either study commenced, an instructor manual was produced, and training workshops for all prospective instructors were held to standardize implementation. Once trained, the same instructor could conduct all major intervention activities. The manual specified the principal goals of each intervention but gave instructors some flexibility in exactly how to achieve these.

#### Efficacy study

Conventional health education in the *Efficacy Study* control group entailed periodic telephone calls (~3 monthly) by local research site staff to offer participants health education and advice (the intervention group did not receive such calls). The multidomain intervention was adapted from that used in the Multidomain Alzheimer's Preventive Trial (MAPT),^27^ which integrated physical exercise, cognitive training, and nutritional counselling components that were straightforward to organize in community settings and well accepted by participants.

The *Efficacy Study* programme scheduled four structured 2 h training sessions in the first month, two during the second, and one in each of the next 10 months (Supporting Information, *Figure* *S1*); the first was held on 30 August 2014. To promote effective delivery, each cluster was divided into smaller groups of five to eight people per session, and research staff made reminder telephone calls to local participants before each session to maximize attendance. The routine curriculum comprised 45 min of physical fitness activities, specifically aerobic exercises, resistance work, and balance and flexibility training; 1 h of cognitive training, including reasoning and memory exercises; and 15 min of general nutrition advice, including a balanced diet and adequate protein intake (Supporting Information, *Appendix* *S1*). Participants were actively encouraged to practice on their own at home. In addition, every 3 or 4 months, some activities were curtailed, and a visiting doctor instead gave a 30–60 min class on preventing/managing chronic disease, which included education about healthy aging, dementia, cardiovascular risk factors, osteoporosis, and sarcopenia.

#### Empowerment study

The enhanced multidomain intervention programme in the *Empowerment Study* replicated the format and schedule of the first 6 months (10 sessions) of the *Efficacy Study* (Supporting Information, *Figure* *S1*). However, the training sessions used new teaching materials, revised and simplified from the standard multidomain prototype, which addressed feedback from a needs‐assessment survey in local communities. Participants were also given a pedometer and post‐curriculum learning sheets to support goal setting and monitoring and additionally empowered by community leader involvement, group competitions, and individual motivation (Supporting Information, *Appendix* *S1*).

### Assessments and outcomes

Baseline demographic and health‐related data included participants' age, sex, race, tobacco smoking and alcohol consumption behavior, and self‐reported history of hypertension, diabetes mellitus, cardiovascular disease, stroke, and/or malignancy. Physical, cognitive, and functional performance were assessed at baseline and 6 months in both studies, with final follow‐up at 12 months in the *Efficacy Study* and at 9 months (3 months after the intervention ceased) in the *Empowerment Study*. The last study assessment was on 9 May 2017, and data were locked on 6 June 2017.

Physical measurements included time taken to walk 6 m at normal walking pace, handgrip strength by dynamometry (Smedlay's Dynamo Meter, TTM, Tokyo, Japan), and physical activity in units of metabolic equivalent of task (MET),^28^ based on a validated Leisure‐Time Physical Activity questionnaire.^29^ Frailty was defined according to modified Cardiovascular Health Study (CHS) criteria,^12^ comprising weak grip of <26.0 kg in men or <18.0 kg in women; walking slower than 0.8 m/s; self‐reported exhaustion on more than 3 days/week; unintentional weight loss of >5.0 kg or 10% during the past year; and physical activity <3.75 MET/h in men or <2.5 MET/h in women (lowest quintile of sex‐specific baseline values). People fulfilling three or more criteria were classed as frail, those who met one or two as prefrail, and those with no such deficits as robust.

General cognitive performance was evaluated using a version of the Montreal Cognitive Assessment screening tool, with cut‐offs adjusted for Taiwanese Chinese users (MoCA_adj_); one point was added to participants educated for <12 years.^30^ The full MoCA battery covers most domains affected by mild cognitive impairment, including visuospatial executive, naming, concentration, language, abstract thinking, delayed recall, and orientation. Functional status was based on established indicators—the five‐item Geriatric Depression Scale (GDS‐5),^31^ Mini‐Nutritional Assessment short‐form (MNA‐SF),^32^ and IADL.^33^

#### Primary and secondary outcomes

The primary outcomes were changes from baseline in CHS frailty score, gait speed, grip strength, and MoCA_adj_. Secondary outcomes were IADL, nutrition status, and depressive symptoms; MNA‐SF ≤11 and/or GDS‐5 ≥2 defined high risk of being malnourished and/or depressed, respectively.

### Statistical analysis

All statistical analyses used SPSS Version 24.0 for Microsoft Windows 7 (IBM Corp., Armonk, NY, USA). Sample size calculations were based on another investigation of the effect of nutritional, cognitive, and physical interventions on frailty,^14^ in which 1 year CHS frailty scores of 1.2 vs. 1.6 in intervention and control groups, respectively, resulted in a Cohen's *d* effect size of 0.39.^34^ Assuming equal cluster sizes, constrained to 20 people on average, and an intra‐cluster correlation coefficient of 0.1, at least 16 clusters in the intervention and control groups would be needed to achieve discriminatory power of 0.8 at the two‐sided alpha level of 0.05; however, anticipating a completion rate of around 70%,^22^, ^23^, ^24^ we aimed to include ≥20 clusters in each group. The analytic population samples included all intervention participants with at least one post‐baseline observation (modified intention to treat). Missing data were not imputed. A generalized linear mixed model, which assumed data to be missing at random, was used to analyze changes in outcome variables as functions of treatment group, time, and Group × Time interaction, with random effect applied at cluster level to account for participant clustering within each community. These analyses were adjusted for statistically significant differences in baseline characteristics between intervention groups, except for significantly correlated pairs of variables, in which case only one was adjusted to avoid collinearity. Analyses were repeated for participants aged ≥75 years. Cohen's *d* effect sizes were calculated from mean changes derived from the generalized linear mixed model.

## Results

### Participant disposition and characteristics

Between 12 February 2014 and 5 May 2016, 1907 people ≥65 years old from 54 community center/neighborhood clusters across five regions of Taiwan were screened for participation in two complementary studies; 1522 (79.8%), all Chinese/Taiwanese, fulfilled eligibility criteria and consented to enroll (*Figure* 1). The *Efficacy Study* assigned 19 clusters (549 participants) to receive multidomain intervention and 21 clusters (533 participants) to conventional health education. The median cluster size was 26. Participants in all 40 clusters received the treatment allocated, and more than 70% completed the study; 25.3% in the intervention group and 29.6% in the control group discontinued or were lost to follow‐up (*Figure* 1A). The *Empowerment Study* separately enrolled another 440 participants, and randomized seven clusters (210 participants) to receive the standard multidomain intervention used in the *Efficacy Study*, and seven clusters (230 participants) to receive the adapted version of the standard prototype, enhanced to empower participants. The median cluster size in the *Empowerment Study* was 33, and the completion rate was ~90% (*Figure* 1B).

Both randomization groups in either study had broadly similar baseline characteristics (*Table* 1). Participants were predominantly women, with average age ≥75 years, and were evenly distributed between rural and urban residents. Half of both study cohorts had high blood pressure, and 20–25% had diabetes or cardiovascular disease. Frailty status and other physical assessments were mostly similar between groups; however, some differences in baseline cognitive performance and IADL were statistically significant. *Efficacy Study* multidomain intervention recipients had better overall cognitive, visuospatial executive, and language performance vs. controls and higher mean IADL (all *P* < 0.05). *Empowerment Study* participants in the enhanced multidomain intervention group performed worse than controls in cognitive domains of naming, concentration, and abstract thinking (all *P* < 0.05).

**Table 1:** Baseline characteristics of the *Efficacy Study* and *Empowerment Study* participant groups

Characteristics: data show mean ± SD [number]^a^ or number/total^a^ (%)		*Efficacy Study*			*Empowerment Study*		
		Total (*n* = 1082)	Health education (*n* = 533)	Standard multidomain (*n* = 549)	Total (*n* = 440)	Standard multidomain (*n* = 210)	Enhanced multidomain (*n* = 230)
**Demographic and health factors**							
Age (years)	All	75.1 ± 6.3	74.9 ± 6.2	75.3 ± 6.4	75.9 ± 7.1	76.3 ± 7.6	75.5 ± 6.6
	≥75	532 (49.2)	262 (49.2)	270 (49.3)	236 (53.6)	111 (52.9)	125 (54.3)
Sex (female)		740 (68.7)	332 (62.4)	408 (74.5)^b^	368 (83.6)	177 (84.3)	191 (83.0)
Education (years)		6.6 ± 4.5	6.4 ± 4.4	6.7 ± 4.5	6.2 ± 4.7	7.1 ± 4.8	5.3 ± 4.5^b^
Urban residence		529 (48.9)	247 (46.3)	282 (51.4)	215 (48.9)	100 (47.6)	115 (50.0)
Current tobacco smoker		58 (5.4)	41 (7.7)	17 (3.1)^b^	5 (1.1)	1 (0.5)	4 (1.7)
Current alcohol consumer		155 (14.3)	83 (15.6)	72 (13.1)	40 (9.1)	20 (9.5)	20 (8.7)
**Medical history** (self‐reported)							
Hypertension		556 (51.4)	266 (49.9)	290 (52.8)	231 (52.6)	103 (49.3)	128 (55.7)
Diabetes mellitus		256 (23.7)	135 (25.3)	121 (22.0)	94 (21.4)	48 (22.9)	46 (20.0)
Cardiovascular disease		232 (21.4)	123 (23.1)	109 (19.9)	102 (23.2)	50 (23.8)	52 (22.6)
Stroke		41 (3.8)	15 (2.8)	26 (4.7)	14 (3.2)	10 (4.8)	4 (1.7)
Malignancy		39 (3.6)	18 (3.4)	21 (3.8)	17 (3.9)	7 (3.3)	10 (4.3)
**Cognitive performance**							
MoCA_adj_		20.1 ± 5.8	19.8 ± 5.5	20.5 ± 5.8^b^	19.9 ± 6.4 [435]	20.4 ± 6.5 [205]	19.4 ± 6.4
Visuospatial executive		2.6 ± 1.7	2.5 ± 1.7	2.7 ± 1.7^b^	2.7 ± 1.6	2.8 ± 1.7	2.7 ± 1.6
Naming		2.3 ± 0.9	2.3 ± 0.9	2.4 ± 0.9	2.0 ± 1.1 [435]	2.2 ± 1.0 [205]	1.9 ± 1.1^b^
Concentration		4.4 ± 1.6	4.4 ± 1.6	4.4 ± 1.6	4.4 ± 1.7	4.6 ± 1.6	4.2 ± 1.7^b^
Language		1.7 ± 1.0	1.6 ± 1.0	1.8 ± 1.0^b^	1.7 ± 1.0	1.7 ± 1.0	1.7 ± 0.9
Abstract		0.7 ± 0.8	0.6 ± 0.8	0.7 ± 0.8	0.6 ± 0.8	0.7 ± 0.8	0.6 ± 0.7^b^
Delayed recall		2.3 ± 1.7	2.2 ± 1.6	2.3 ± 1.7	2.3 ± 1.8	2.4 ± 1.9	2.2 ± 1.7
Orientation		5.3 ± 1.2	5.3 ± 1.1	5.4 ± 1.1	5.1 ± 1.3	5.1 ± 1.3	5.1 ± 1.2
**Physical performance**							
Frailty status	Robust	374/1058 (35.3)	177/526 (33.7)	197/532 (37.0)	184/425 (43.3)	81/203 (39.9)	103/222 (46.4)
	Prefrail	605/1058 (57.2)	307/526 (58.4)	298/532 (56.0)	207/425 (48.7)	109/203 (53.7)	98/222 (44.1)
	Frail	79/1058 (7.5)	42/526 (8.0)	37/532 (7.0)	34/425 (8.0)	13/203 (6.4)	21/222 (9.5)
	CHS frailty score	1.0 ± 1.0 [1058]	1.1 ± 1.0 [526]	1.0 ± 1.0 [532]	0.9 ± 1.0 [425]	0.9 ± 0.9 [203]	0.9 ± 1.0 [222]
Handgrip strength (kg)		22.7 ± 8.3 [1075]	23.1 ± 9.0 [530]	22.3 ± 7.6 [545]	21.9 ± 7.0 [436]	21.7 ± 6.9 [208]	22.0 ± 7.2 [228]
Gait speed (m/s)		1.0 ± 0.3 [1066]	0.9 ± 0.3 [528]	1.0 ± 0.3 [538]	1.1 ± 0.3 [435]	1.1 ± 0.4 [208]	1.1 ± 0.3 [227]
Physical activity (MET)		14.3 ± 16.9 [1077]	14.9 ± 18.5 [532]	13.7 ± 15.1 [545]	15.3 ± 17.3 [438]	15.5 ± 17.9 [209]	15.1 ± 16.7 [229]
**Functional assessments**							
GDS‐5		0.4 ± 0.9 [1079]	0.4 ± 0.9	0.4 ± 0.9 [546]	0.4 ± 0.8	0.4 ± 0.8	0.4 ± 0.8
Depressive mood (GDS‐5 ≥2)		96 (8.9)	45 (8.4)	51 (9.3)	39 (8.9)	20 (9.5)	19 (8.3)
Nutrition status (MNA‐SF)		13.2 ± 1.2 [1073]	13.2 ± 1.2 [532]	13.1 ± 1.3 [541]	13.3 ± 1.0 [433]	13.2 ± 1.0 [208]	13.3 ± 1.0 [225]
Malnutrition (MNA‐SF ≤11)		96/1073 (8.9)	41/532 (7.7)	55/541 (10.2)	29/433 (6.7)	16/208 (7.7)	13/225 (5.8)
Instrumental ADL		7.3 ± 1.2 [1077]	7.2 ± 1.2	7.4 ± 1.1^b^ [544]	7.5 ± 1.1	7.5 ± 1.1	7.4 ± 1.2

ADL, activities of daily living; CHS, Cardiovascular Health Study; GDS‐5, five‐item Geriatric Depression Scale; MET, metabolic equivalent of task; MNA‐SF, Mini‐Nutritional Assessment short‐form; MoCA_adj_, Montreal Cognitive Assessment (adjusted cut‐off).

a If fewer than the entire group.

b Statistically significant between‐group difference by Student's *t*‐test.

### Intervention outcomes

#### Efficacy study: standard multidomain intervention

**Figure 2:**
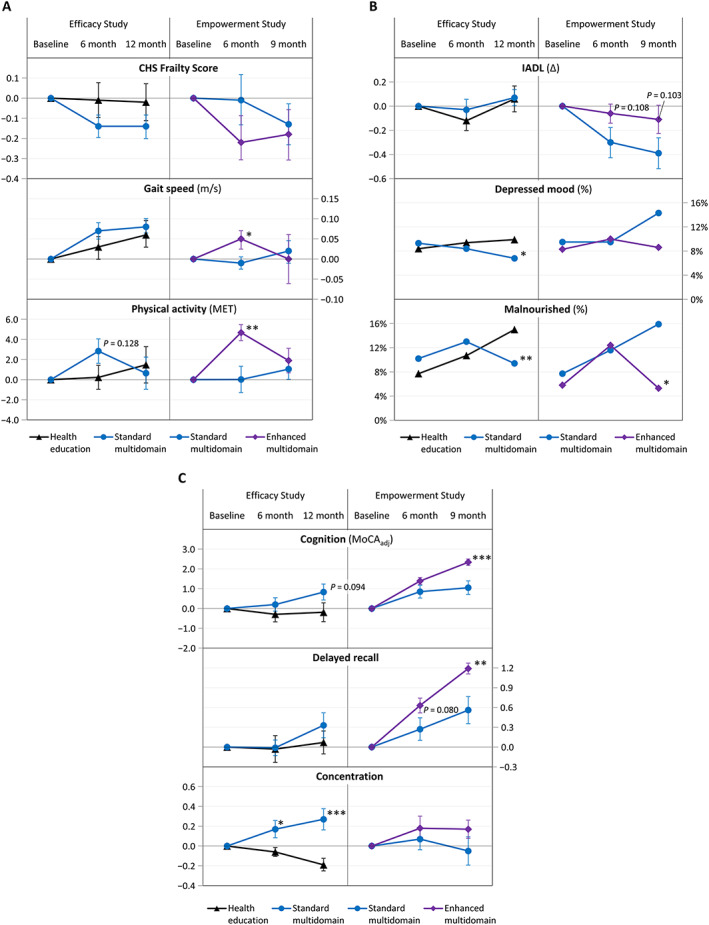
Mean changes from baseline performance. (A) Physical domains; (B) functional domains; and (C) cognitive domains. CHS, Cardiovascular Health Study; MET, metabolic equivalent of task; IADL, instrumental activities of daily living; MoCA_adj_, Montreal Cognitive Assessment (adjusted cut‐off). ^*^ *P* < 0.05; ^**^ *P* < 0.01; ^***^ *P* < 0.001; vertical bars indicate standard error.

**Figure 3:**
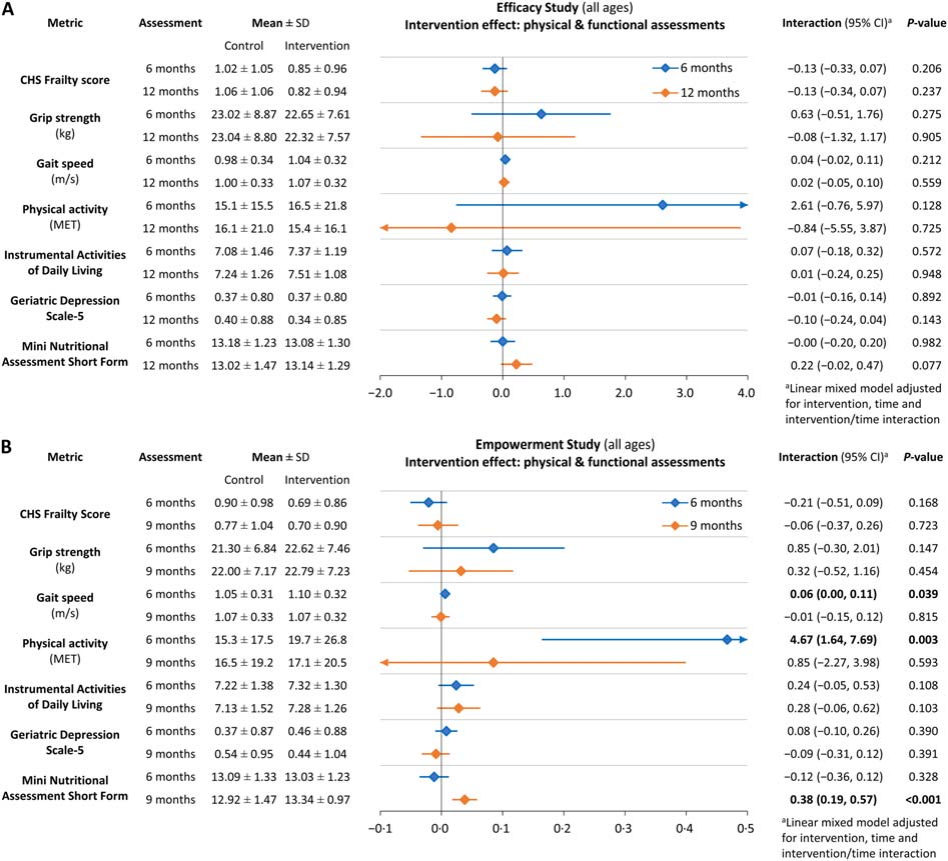
Intervention effects on physical and functional performance. (A) *Efficacy Study*; (B) *Empowerment Study*. CHS, Cardiovascular Health Study; MET, metabolic equivalent of task. Horizontal bars indicate 95% confidence intervals at 6 months (blue) and 12 or 9 months (orange).

**Figure 4:**
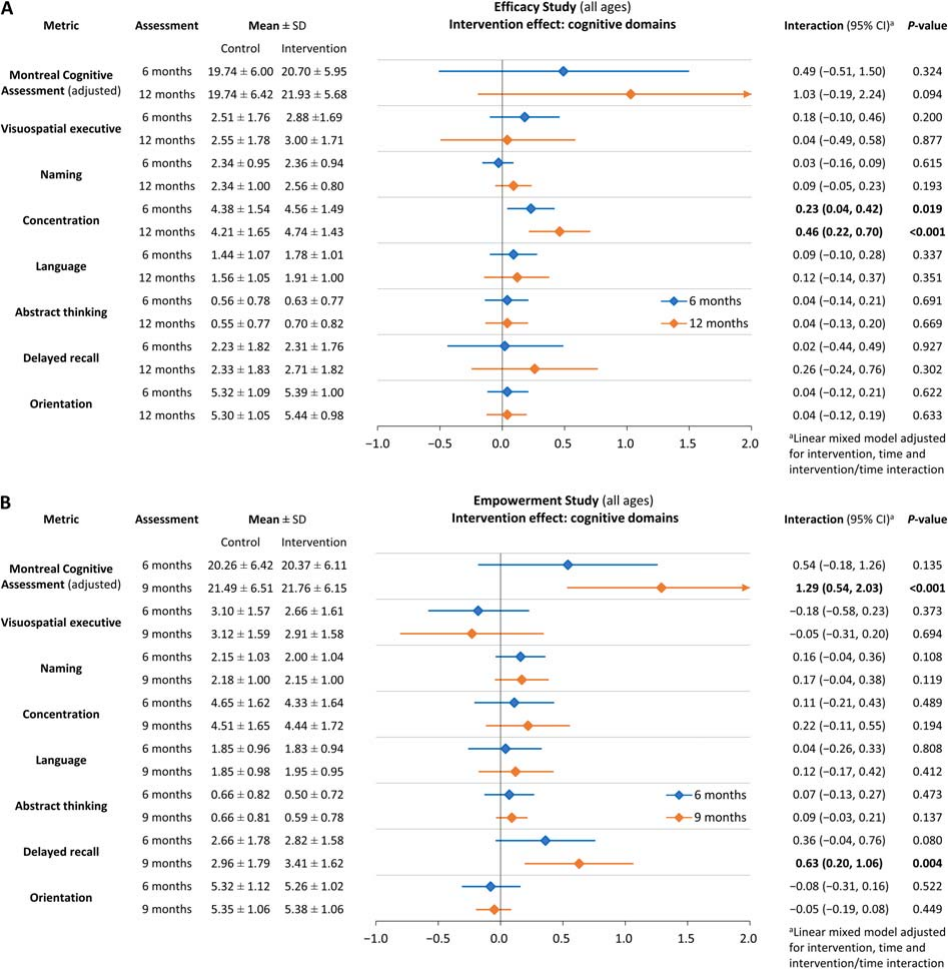
Intervention effects on cognitive performance. (A) *Efficacy Study*; (B) *Empowerment Study*. Horizontal bars indicate 95% confidence intervals at 6 months (blue) and 12 or 9 months (orange).

Although participants in the *Efficacy Study* multidomain intervention had lower CHS frailty scores at interim and final follow‐up than at baseline and compared with controls (*Figure* 2A; Supporting Information, *Table* *S1*), neither difference was statistically significant. The multidomain intervention did not significantly improve other overall physical or functional outcomes by 6 months, but concentration improved significantly (*Figures* 2A–2C, 3A, and 4A; Supporting Information, *Tables* *S1* and *S2*). At 12 months, intervention group participants were half as likely than controls to have depressed mood [odds ratio 0.56, 95% confidence interval (CI) 0.32, 0.99, *P* = 0.044] or malnutrition (odds ratio 0.45, 95% CI 0.26, 0.78, *P* = 0.004); continued gains in concentration contributed to a rising trend in overall cognitive performance (MoCA_adj_ 1.03, 95% CI −0.19, 2.24, *P* = 0.094) (*Figures* 2B and 2C and 4A; Supporting Information, *Tables* *S1* and *S2*).

#### Empowerment study: enhanced multidomain intervention

The results of enhanced vs. standard multidomain intervention in the *Empowerment Study* had commonalities with the intervention effects observed in the *Efficacy Study*, but with significantly improved gait speed and physical activity at 6 months, and even lower prevalence of malnutrition (odds ratio 0.39, 95% CI 0.18, 0.84, *P* = 0.016) and enhanced delayed recall and overall cognitive performance at 9 months (*Figures* 2A–2C, 3B, and 4B; Supporting Information, *Tables S1* and *S2*). Unlike the *Efficacy Study*, depressed mood did not differ significantly between the intervention and control groups (*Figure* 2B; Supporting Information, *Table S1*).

#### ≥75‐year‐olds

The prevalence of frailty was higher among study participants aged ≥75 years compared with the entire population: 10.5% vs. 7.6% overall; 9.2% vs. 7.5% in the *Efficacy Study*; and 13.3% vs. 7.8% in the *Empowerment Study*. *Efficacy Study* participants ≥75 years old had some more pronounced improvements relative to controls than those observed overall, including significantly lower CHS frailty scores at both 6 and 12 months, stronger grip at 6 months, and lower GDS‐5 score and enhanced delayed recall and overall cognition at 12 months (Supporting Information, *Tables* *S1* and *S2* and *Figures* *S2*, *S3*, and *S4*); ≥75‐year‐olds in the *Empowerment Study* had a significantly lower frailty score and less prevalent frailty at 6 months and more pronounced differences in physical activity and IADL at both 6 and 9 months. Both the standard and enhanced multidomain interventions significantly improved cognitive performance among ≥75‐year‐olds, with a sustained gain 3 months after the participant‐empowered intervention ceased similar to that seen in the overall population (Supporting Information, *Tables* *S1* and *S2* and *Figures* *S2*, *S3*, and *S4*).

### Estimated magnitude of intervention effects

Estimated effect sizes in direct comparisons between each intervention and its control at 6 and 12 months (*Efficacy Study*) or 9 months (*Empowerment Study*) were congruent with the significant interactions identified in linear mixed model analyses (Supporting Information, *Table* *S3*). Cohen's *d* coefficients indicated small positive effects on CHS frailty score, physical activity, delayed recall, and IADL at 6 months in the *Empowerment Study* and on concentration at 12 months in the *Efficacy Study*. Similar effect sizes on overall cognitive performance, delayed recall, IADL, and malnutrition were evident at the 9 month follow‐up in the *Empowerment Study*. Because each study involved different participants, it was not possible to estimate the effect of enhanced multidomain intervention relative to conventional health education directly; however, indirect comparison indicated small/medium overall effects on CHS frailty score, gait speed, physical activity, delayed recall, and IADL at 6 months and on cognitive performance, IADL, and depression at study end, with stronger effects on delayed recall and malnutrition.

## Discussion

The Taiwan Health Promotion Intervention Study for Elders has produced further evidence that pragmatic multidomain interventions may simultaneously help to reverse both physical and cognitive decline among vulnerable older people. Importantly, an enhanced programme that empowered and motivated participants produced more pronounced benefits, and this is the first study showing that participants ≥75 years old had even greater improvements in their physical and mental performance than younger ones, including significantly diminished prevalence of frailty during the intervention. The interventions were straightforward to implement in the community setting by trained personnel. These results support a rationale for universal implementation of community‐based programmes to promote healthy aging and reduce late‐life disability and cognitive decline, and have important implications for preparatory policy planning.

The standard multidomain intervention in our *Efficacy Study* resulted in improvements across core components of phenotypic frailty, particularly in physical (grip strength) and functional (depression and malnutrition) domains, with consequently reduced CHS frailty scores. Significantly improved IADL among ≥75‐year‐olds both during and after the enhanced multidomain intervention, with a less pronounced effect in the *Efficacy Study*, suggests that reinforcing lifestyle behavior changes could potentially pre‐empt, even reverse, disability. Malnutrition is another important determinant of frailty and late‐life cognitive decline,^21^ and both multidomain interventions had significantly lessened the prevalence of malnutrition at final follow‐up.

The improvement in physical activity in the *Efficacy Study* was greater over 0–6 months than 0–12 months, which may imply that decreased intervention frequency attenuated the effect; on the other hand, improvements in cognition, depressed mood, and malnutrition were greater after 12 months than at 6 months. Likewise, gains in gait speed and physical activity plateaued from 6 months, after the *Empowerment Study* intervention ended, whereas improvements in nutrition status and cognition were sustained at the 9 month post‐intervention follow‐up. Later‐onset improvements in cognitive performance and nutrition status after the intervention intensity dropped to once‐monthly maintenance session, or ceased in the *Empowerment Study*, suggest that a pragmatic community‐based programme could yield sustainable benefits. Pertinently, a less onerous intervention schedule would be more amenable to national‐scale implementation.

Further studies are warranted to determine for how long the legacy effect of participant empowerment persists, and to follow‐up longer‐term outcomes such as quality of life, which may be a more appropriate indicator of healthy aging, or even mortality.

These findings reinforce strong evidence that interventions that incorporate exercise training, either with or without nutrition, are effective in reversing frailty and physical disability, with some sustained gains.^14^, ^15^, ^20^, ^35^, ^36^, ^37^, ^38^, ^39^ In contrast, several geriatric care models that implemented individual needs assessment and tailored multidisciplinary management have shown little or no benefit compared with usual primary care.^15^, ^22^, ^23^, ^24^, ^25^ Disparity between the results of participatory vs. service‐based approaches may reflect the importance of the psychosocial context in interventions that improve well‐being, which has been underappreciated.^15^, ^18^

In long‐running controversy about how best to define and operationalize the elusive concept of frailty, proponents of cumulative deficit models, which integrate cognitive and psychosocial dimensions among others, have criticized ‘biological’ frailty as narrow and simplistic.^4^, ^21^ However, phenotypic criteria are practical to apply, and fewer intervention studies have employed multidimensional definitions of frailty.^15^, ^21^ A notable feature of successful interventions such as ours is that they provide participants with opportunities for social inclusion and mental stimulation, such as group exercise, cognitive training, or psychological support.^14^, ^20^, ^35^, ^37^ Even better results may be obtained by engaging, empowering, and motivating participants, for example, by goal setting.^37^, ^40^ In the *Empowerment Study*, such enhancements consolidated gains in physical activity, improved nutrition status, and overall cognition; the remarkable 90% completion rate indicates an unusually high level of participant satisfaction and may also be attributed to the peer effect in a cluster‐randomized study design. Although the effect sizes we detected were small/medium at best, small long‐term effects can nonetheless be highly consequential in a public health context.^41^ Furthermore, indirect comparison of the *Efficacy* and *Empowerment Studies* suggested an additive effect of enhanced multidomain intervention, possibly reflecting functional interrelationships between physical activity, cognition, and frailty.

Physical exercise probably has psychological benefits, and cognitive training, vice versa, appears to improve aspects of physical function such as balance and gait speed, although the mechanisms remain obscure.^14^, ^16^ Despite evidence that cognitive reserve may support coping in older age and that cognitive deficits or mood disturbances may be other manifestations of frailty,^4^, ^21^ few studies have evaluated cognitive or mental health outcomes.^15^ A multidomain intervention targeting vascular risk factors for dementia had no effect on cognition or depressive symptoms, nor in preventing dementia.^24^ In the Lifestyle Interventions and Independence for Elders study, a 2 year programme of moderate‐intensity physical exercise did not improve cognitive functioning, but neither did it decline.^42^ However, a multidomain intervention that included diet, exercise, and cognitive training, as well as fostering social activities, reduced the risk of cognitive decline in at‐risk older people.^41^ In an ancillary MAPT subgroup study, intervention with omega‐3 supplementation and/or physical activities, cognitive exercises and nutritional advice, improved cognitive performance compared with placebo among elderly people at risk for developing dementia who had positive amyloid status.^43^ The MAPT multidomain intervention was also associated with reduced risk of incident frailty in a secondary analysis.^44^ In Singapore, cognitive training focused on short‐term memory, attention, information processing and reasoning, reduced frailty, and improved lower limb strength.^14^ Our standard multidomain intervention significantly improved concentration, which, combined with delayed recall, contributed to enhancing overall cognitive performance among ≥75‐year‐olds; borderline improvement of MoCA_adj_ at 12 months in the whole *Efficacy Study* cohort was possibly due to insufficient follow‐up. The multidomain intervention also significantly reduced the prevalence of depressed mood. The enhanced multidomain intervention did significantly improve overall cognition, despite a non‐significant between‐group effect on concentration, driven by significantly improved delayed recall relative to the standard multidomain intervention.

Frailty interventions have been shown to be effective at ages from 65 years upward, but the potential effect of participant's age on their impact is little studied. Among four reports,^15^ only one found age to be a moderating factor, with younger subjects more likely to revert from frail to robust status.^45^ In both of our studies, ≥75‐year‐olds, who had higher prevalence of frailty, appeared to have some more pronounced and sustained improvements in domains of physical performance (CHS frailty score and handgrip strength), functioning (IADL), and overall cognition (MoCA_adj_) than younger participants. This novel finding suggests that people at potentially higher risk for adverse consequences of frailty may benefit most from such intervention, in which case healthy aging initiatives that focus on the older elderly may be particularly expedient.

The Taiwan Health Promotion Intervention Study for Elders used a rigorous, evidence‐based, cluster‐randomized design to produce data commensurate with those of other randomized controlled trials and thereby contribute to efforts to ascertain the most effective approaches to preventing or treating frailty, which is urgent given its increasing socio‐economic impact.^19^ Although cluster‐randomized trials are relatively scarce compared with typical randomized designs, perhaps due to being hard to conduct in real‐world settings, this powerful approach is increasingly common in modern pragmatic trials. We recruited a nationally representative sample of prefrail/frail individuals from rural and urban areas throughout Taiwan; broadly similar baseline characteristics between intervention arms indicate that cluster randomization did not introduce significant bias and that the impact of unmeasured confounders may have been minimized. Another strength of these studies was extraordinarily high retention rates, especially in empowered participants, which highlights the value of considering the characteristics and needs of potential participants when designing community‐based intervention programs. However, this may also reflect unique local circumstances, as noted in another study of ethnically Chinese older adults, and not necessarily extrapolate to all prefrail populations.^14^ Cost‐effectiveness will be a key consideration in incorporating such interventions into healthy aging strategies, and in this regard, participatory communal programs may provide better value for money than individualized treatment, especially for very frail individuals.^15^, ^37^ Our study provides a rare and powerful example of a practical and sustainable community‐based intervention that can be implemented using simple materials and existing facilities. Although the Taiwan Health Promotion Intervention Study for Elders was not intended to evaluate cost‐effectiveness, the interventions were designed to be affordable, in not necessarily requiring any specialist medical equipment or facilities.

Notwithstanding these strengths, we acknowledge several limitations. (i) Participant blinding was difficult to achieve in the *Efficacy Study*, due to the evident difference in the interventions compared, but this issue is inherent to behavior‐based studies. (ii) The *Empowerment Study*, with only seven clusters per arm, was probably underpowered. (iii) The study design precluded direct comparison of the effect of participant‐empowered multidomain intervention vs. conventional health education, or evaluation of the contribution of individual multidomain components to reducing frailty; however, the exigent knowledge gap is not whether, but which, multidomain intervention is better.

Overall, our results affirm that an integrated multidomain intervention programme, including physical exercise, cognitive training, nutrition advice, and disease education, can prevent or reverse frailty by improving principal determinants of physical well‐being and mental health among prefrail/frail community‐dwelling older people, especially among people older than 75. The standardized protocol used in this study is amenable to inclusion in policies to promote healthy aging and may supplement them more effectively and sustainably, if implemented via strategies that motivate and empower participants.

## Conflict of interest

L.‐K.C., A.‐C.H., W.‐J.L., L.‐N.P., M.‐H.L., S.‐F.S., C.‐H.L., and S.‐T.C. declare no conflicts of interest. D.L.N. is a professional medical writer employed at time of writing by Full Universe Integrated Marketing Ltd., Taiwan.

## Ethical standards

The authors certify that they comply with the ethical guidelines for publishing in the *Journal of Cachexia, Sarcopenia and Muscle*.^46^

## Supporting information

Table S1. Changes in participants' physical performance and functional status during the *Efficacy Study* and during/after the *Empowerment Study*

Table S2. Changes in participants' cognitive performance domains during the *Efficacy Study* and during/after the *Empowerment Study*

Table S3. Combined effect of interventions on cognitive, physical, and function domains at 6 months

Appendix S1. *Efficacy and Empowerment Studies*: standard and enhanced multidomain intervention protocols

Figure S1. *Efficacy* and *Empowerment Studies* intervention and assessment schemes

Figure S2. Mean changes from baseline performance among participants ≥75 years old

Figure S3. Intervention effects on physical and functional performance among participants ≥75 years old

Figure S4. Intervention effects on cognitive performance among participants ≥75 years old
