# Supplementary material for: Efficacy of multidomain interventions to improve physical frailty, depression and cognition: data from cluster‐randomized controlled trials
Source: J Cachexia Sarcopenia Muscle. 2020 Mar 5;11(3):650–62. doi: 10.1002/jcsm.12534 (PMC7296266; doi:10.1002/jcsm.12534)
Supplement: Supplementary file 1 — Table S1. Changes in participants' physical performance and functional status during the Efficacy Study and during/after the Empowerment Study [file JCSM-11-650-s006.pdf]

Table S1 Changes in participants' physical performance and functional status during the *Efficacy Study* and during/after the *Empowerment Study*

Outcome metrics	Assessments: Mean ± SD (*N*)	*Efficacy Study*: Intervention for 12 months				Assessments at baseline, 6 & 12 months				*Empowerment Study*: Intervention for 6 months				Assessments at baseline, 6 & 9 months			
		Health education		Standard multidomain		Interaction (95% CI)[a,b]		P-value		Standard multidomain		Enhanced multidomain		Interaction (95% CI)[a,b]		P-value	
		All	≥75 Y	All	≥75 Y	All	≥75 Y	All	≥75 Y	All	≥75 Y	All	≥75 Y	All	≥75 Y	All	≥75 Y
**CHS Frailty Score**	Baseline	1.06 ± 0.95<br>(526)	1.26 ± 0.93<br>(259)	0.99 ± 0.97<br>(532)	1.23 ± 0.96<br>(261)					0.90 ± 0.92<br>(203)	1.22 ± 0.99<br>(105)	0.89 ± 1.03<br>(222)	1.10 ± 1.12<br>(120)				
	6 months	1.02 ± 1.05<br>(417)	1.28 ± 1.13<br>(204)	0.85 ± 0.96<br>(469)	1.07 ± 1.04<br>(232)					0.90 ± 0.98<br>(187)	1.27 ± 1.06<br>(98)	0.69 ± 0.86<br>(200)	0.85 ± 0.94<br>(102)				
	6-month Δ<br>(95% CI)	–0.01<br>(–0.18, 0.16)	0.07<br>(–0.08, 0.23)	–0.14<br>(–0.25, –0.03)	–0.16<br>(–0.31, –0.02)	–0.13<br>(–0.33, 0.07)	**–0.24**<br>**(–0.45, –0.03)**	0.206	**0.025**	–0.01<br>(–0.25, 0.24)	0.05<br>(–0.17, 0.28)	–0.22<br>(–0.39, –0.04)	–0.28<br>(–0.52, –0.05)	–0.21<br>(–0.51, 0.09)	**–0.34**<br>**(–0.66, –0.01)**	0.168	**0.044**
	Study ends at<br>12 or 9 months	1.06 ± 1.06<br>(364)	1.39 ± 1.13<br>(176)	0.82 ± 0.94<br>(403)	1.06 ± 1.00<br>(192)					0.77 ± 1.04<br>(189)	1.20 ± 1.16<br>(97)	0.70 ± 0.90<br>(205)	0.86 ± 0.99<br>(105)				
	Study-end Δ<br>(95% CI)	–0.02<br>(–0.20, 0.16)	0.10<br>(–0.11, 0.31)	–0.14<br>(–0.26, –0.03)	–0.18<br>(–0.37, 0.00)	–0.13<br>(–0.34, 0.07)	**–0.28**<br>**(–0.56, 0.00)**	0.237	**0.044**	–0.13<br>(–0.33, 0.07)	–0.03<br>(–0.27, 0.21)	–0.18<br>(–0.43, 0.06)	–0.25<br>(–0.45, –0.05)	–0.06<br>(–0.37, 0.26)	–0.22<br>(–0.53, 0.09)	0.723	0.170
**CHS Frailty prevalence** [n (%)]	Baseline	42 (8.0)<br>(526)	26 (10.0)<br>(259)	37 (7.0)<br>(532)	22 (8.4)<br>(261)					13 (6.4)<br>(203)	12 (11.4)<br>(105)	21 (9.5)<br>(222)	18 (15.0)<br>(120)				
	6 months	44 (10.6)<br>(417)	32 (15.7)<br>(204)	31 (6.6)<br>(469)	22 (9.5)<br>(232)	0.68<br>(0.34, 1.36)	0.63<br>(0.25, 1.55)	0.279	0.310	17 (9.1)<br>(187)	16 (16.3)<br>(98)	8 (4.0)<br>(200)	6 (5.9)<br>(102)	NA	**0.21**<br>**(0.06, 0.73)**	NA	**0.014**
	Study ends at<br>12 or 9 months	37 (10.2)<br>(364)	28 (15.9)<br>(176)	25 (6.2)<br>(403)	17 (8.9)<br>(192)	0.70<br>(0.34, 1.42)	0.60<br>(0.24, 1.48)	0.316	0.264	15 (7.9)<br>(189)	13 (13.4)<br>(97)	8 (4.4)<br>(205)	8 (7.6)<br>(105)	NA	0.38<br>(0.13, 1.15)	NA	0.087
**Handgrip strength** [kg]	Baseline	23.13 ± 8.96<br>(530)	21.78 ± 9.14<br>(260)	22.30 ± 7.60<br>(545)	20.77 ± 6.78<br>(267)					21.66 ± 6.87<br>(208)	19.86 ± 6.94<br>(110)	22.03 ± 7.22<br>(228)	20.15 ± 5.88<br>(124)				
	6 months	23.02 ± 8.87<br>(421)	21.55 ± 9.24<br>(205)	22.65 ± 7.61<br>(488)	21.04 ± 6.86<br>(243)					21.30 ± 6.84<br>(189)	19.58 ± 6.69<br>(100)	22.62 ± 7.46<br>(201)	20.62 ± 6.77<br>(103)				
	6-month Δ<br>(95% CI)	–0.23<br>(–1.22, 0.76)	–0.62<br>(–1.33, 0.10)	0.39<br>(–0.17, 0.96)	0.44<br>–0.27, 1.14)	0.63<br>(–0.51, 1.76)	**1.05**<br>**(0.05, 2.06)**	0.275	**0.039**	–0.34<br>(–1.04, 0.37)	–0.29<br>(–1.04, 0.45)	0.51<br>(–0.43, 1.46)	0.38<br>(–0.85, 1.62)	0.85<br>(–0.30, 2.01)	0.68<br>(–0.75, 2.11)	0.147	0.351
	Study ends at<br>12 or 9 months	23.04 ± 8.80<br>(375)	21.08 ± 8.39<br>(177)	22.32 ± 7.57<br>(408)	20.63 ± 7.03<br>(197)					22.00 ± 7.17<br>(189)	19.84 ± 6.84<br>(97)	22.79 ± 7.23<br>(206)	20.83 ± 6.36<br>(106)				
	Study-end Δ<br>(95% CI)	0.06<br>(–1.01, 1.14)	–0.53<br>(–1.45, 0.40)	–0.01<br>(–0.68, 0.66)	0.29<br>(–0.25, 0.82)	–0.08<br>(–1.32, 1.17)	0.81<br>(–0.26, 1.88)	0.905	0.139	0.31<br>(–0.10, 0.72)	0.04<br>(–0.78, 0.86)	0.63<br>(–0.10, 1.36)	0.49<br>(–0.06, 1.04)	0.32<br>(–0.52, 1.16)	0.45<br>(–0.53, 1.43)	0.454	0.364
	Baseline	0.94 ± 0.34<br>(528)	0.83 ± 0.30<br>(260)	0.97 ± 0.31<br>(538)	0.89 ± 0.29<br>(264)					1.06 ± 0.36<br>(208)	0.92 ± 0.33<br>(109)	1.06 ± 0.30<br>(227)	0.99 ± 0.31<br>(123)				

**Gait speed** [m/s]	6 months	0.98 ± 0.34<br>(420)	0.87 ± 0.30<br>(205)	1.04 ± 0.32<br>(485)	0.96 ± 0.31<br>(241)					1.05 ± 0.31<br>(188)	0.92 ± 0.32<br>(98)	1.10 ± 0.32<br>(201)	1.01 ± 0.31<br>(103)				
	6-month Δ<br>(95% CI)	0.03<br>(−0.03, 0.08)	0.01<br>(−0.04, 0.06)	0.07<br>(0.03, 0.11)	0.07<br>(0.02, 0.12)	0.04<br>(−0.02, 0.11)	0.06<br>(−0.02, 0.13)	0.212	0.130	−0.01<br>(−0.04, 0.02)	0.00<br>(−0.05, 0.05)	0.05<br>(0.00, 0.09)	0.03<br>(−0.01, 0.08)	**0.06<br>(0.00, 0.11)**	0.03<br>(−0.03, 0.10)	**0.039**	0.348
	Study ends at<br>12 or 9 months	1.00 ± 0.33<br>(367)	0.89 ± 0.30<br>(177)	1.07 ± 0.32<br>(405)	0.97 ± 0.31<br>(194)					1.07 ± 0.33<br>(189)	0.91 ± 0.32<br>(97)	1.07 ± 0.32<br>(207)	1.00 ± 0.31<br>(105)				
	Study-end Δ<br>(95% CI)	0.06<br>(0.00, 0.13)	0.06<br>(0.00, 0.12)	0.08<br>(0.05, 0.12)	0.07<br>(0.02, 0.12)	0.02<br>(−0.05, 0.10)	0.00<br>(−0.07, 0.08)	0.559	0.915	0.02<br>(−0.04, 0.07)	0.00<br>(−0.09, 0.08)	0.00<br>(−0.12, 0.12)	0.00<br>(0.06, 0.07)	−0.01<br>(−0.15, 0.12)	0.01<br>(−0.10, 0.11)	0.815	0.884
**Physical activity** [MET]	Baseline	14.9 ± 18.5<br>(532)	13.5 ± 16.8<br>(260)	13.7 ± 15.1<br>(545)	12.8 ± 15.5<br>(269)					15.5 ± 17.9<br>(209)	14.4 ± 18.7<br>(110)	15.1 ± 16.7<br>(229)	12.7 ± 15.6<br>(124)				
	6 months	15.1 ± 15.5<br>(419)	12.9 ± 13.0<br>(205)	16.5 ± 21.8<br>(483)	15.2 ± 19.7<br>(240)					15.3 ± 17.5<br>(190)	12.5 ± 14.8<br>(100)	19.7 ± 26.8<br>(201)	18.5 ± 30.5<br>(103)				
	6-month Δ<br>(95% CI)	0.23<br>(−2.12, 2.58)	−0.78<br>(−3.48, 2.09)	2.84<br>(0.45, 5.23)	2.46<br>(−0.19, 5.13)	2.61<br>(−0.76, 5.97)	3.24<br>(−0.61, 7.09)	0.128	0.099	0.02<br>(−2.56, 2.60)	−2.03<br>(−4.13, 0.07)	4.68<br>(3.11, 6.26)	6.05<br>(3.36, 8.74)	**4.67<br>(1.64, 7.69)**	**8.08<br>(4.64, 11.51)**	**0.003**	**<0.001**
	Study ends at<br>12 or 9 months	16.1 ± 21.0<br>(374)	15.2 ± 24.0<br>(177)	15.4 ± 16.1<br>(408)	14.4 ± 14.4<br>(196)					16.5 ± 19.2<br>(189)	12.9 ± 17.3<br>(97)	17.1 ± 20.5<br>(206)	16.2 ± 24.1<br>(105)				
	Study-end Δ<br>(95% CI)	1.48<br>(−2.06, 5.02)	1.85<br>(−3.18, 6.88)	0.64<br>(−2.50, 3.78)	0.62<br>(−3.55, 4.49)	−0.84<br>(−5.55, 3.87)	−1.23<br>(−7.72, 5.25)	0.725	0.707	1.05<br>(−0.96, 3.07)	−1.57<br>(−3.73 ,0.60)	1.90<br>(−0.49, 4.30)	3.39<br>(−0.20, 6.99)	0.85<br>(−2.27, 3.98)	**4.96<br>(0.77, 9.15)**	0.593	**0.020**
**Geriatric Depression Scale-5**	Baseline	0.37 ± 0.85<br>(533)	0.36 ± 0.85<br>(262)	0.40 ± 0.88<br>(546)	0.44 ± 0.91<br>(270)					0.38 ± 0.78<br>(210)	0.38 ± 0.70<br>(111)	0.38 ± 0.78<br>(230)	0.35 ± 0.69<br>(125)				
	6 months	0.37 ± 0.80<br>(424)	0.44 ± 0.90<br>(207)	0.37 ± 0.80<br>(489)	0.40 ± 0.79<br>(243)					0.37 ± 0.87<br>(190)	0.44 ± 0.94<br>(100)	0.46 ± 0.88<br>(201)	0.46 ± 0.88<br>(103)				
	6-month Δ<br>(95% CI)	−0.01<br>(−0.12, 0.10)	0.08<br>(−0.08, 0.24)	−0.02<br>(−0.12, 0.09)	−0.04<br>(−0.18, 0.11)	−0.01<br>(−0.16, 0.14)	−0.12<br>(−0.33, 0.10)	0.892	0.279	−0.01<br>(−0.14, 0.12)	0.06<br>(−0.16, 0.28)	0.07<br>(−0.06, 0.20)	0.10<br>(−0.12, 0.32)	0.08<br>(−0.10, 0.26)	0.04<br>(−0.27, 0.35)	0.390	0.808
	Study ends at<br>12 or 9 months	0.40 ± 0.88<br>(375)	0.44 ± 0.93<br>(179)	0.34 ± 0.85<br>(409)	0.35 ± 0.82<br>(197)					0.54 ± 0.95<br>(189)	0.61 ± 1.00<br>(97)	0.44 ± 1.04<br>(209)	0.44 ± 0.96<br>(107)				
	Study-end Δ<br>(95% CI)	0.04<br>(−0.05, 0.13)	0.08<br>(0.00, 0.17)	−0.07<br>(−0.17, 0.04)	−0.12<br>(−0.22, -0.01)	−0.10<br>(−0.24, 0.04)	**−0.20<br>(−0.34, −0.06)**	0.143	**0.004**	0.16<br>(−0.02, 0.34)	0.23<br>(−0.01, 0.47)	0.07<br>(−0.05, 0.19)	0.09<br>(−0.07, 0.24)	−0.09<br>(−0.31, 0.12)	−0.14<br>(−0.43, 0.15)	0.391	0.332
**Depressed mood** [n (%) with GDS-5 ≥2]	Baseline	45 (8.4)<br>(533)	22 (8.4)<br>(262)	51 (9.3)<br>(546)	29 (10.8)<br>(270)					20 (9.5)<br>(210)	10 (9.0)<br>(111)	19 (8.3)<br>(230)	8 (6.4)<br>(125)				
	6 months	40 (9.4)<br>(424)	25 (12.4)<br>(207)	41 (8.4)<br>(489)	21 (8.8)<br>(243)	0.81<br>(0.42, 1.58)	0.52<br>(0.20, 1.36)	0.536	0.182	18 (9.5)<br>(190)	12 (12.0)<br>(100)	20 (10.0)<br>(201)	11 (10.7)<br>(103)	1.22<br>(0.58, 2.58)	1.27<br>(0.43, 3.73)	0.596	0.665

	Study ends at 12 or 9 months	37 (9.9) (375)	21 (11.7) (179)	28 (6.8) (409)	15 (7.7) (197)	**0.56 (0.32, 0.99)**	**0.45 (0.24, 0.85)**	**0.044**	**0.014**	27 (14.3) (189)	17 (17.5) (97)	18 (8.6) (209)	8 (7.5) (107)	0.66 (0.30, 1.45)	0.55 (0.21, 1.44)	0.297	0.220
**Mini Nutritional Assessment Short Form**	Baseline	13.22 ± 1.15 (532)	13.16 ± 1.16 (261)	13.12 ± 1.26 (541)	13.08 ± 1.29 (267)					13.24 ± 1.03 (208)	13.10 ± 1.08 (109)	13.28 ± 1.04 (225)	13.18 ± 1.01 (120)				
	6 months	13.18 ± 1.23 (419)	13.01 ± 1.41 (206)	13.08 ± 1.30 (486)	13.04 ± 1.52 (241)					13.09 ± 1.33 (190)	12.87 ± 1.54 (100)	13.03 ± 1.23 (201)	12.95 ± 1.17 (103)				
	6-month Δ (95% CI)	−0.04 (−0.20, 0.11)	−0.15 (−0.36, 0.08)	−0.05 (−0.18, 0.09)	−0.05 (−0.19, 0.10)	0.00 (−0.20, 0.20)	0.10 (−0.16, 0.36)	0.982	0.447	−0.14 (−0.33, 0.05)	−0.23 (−0.55, 0.09)	−0.26 (−0.41, −0.12)	−0.23 (−0.36, −0.09)	−0.12 (−0.36, 0.12)	0.00 (−0.35, 0.35)	0.328	0.999
	Study ends at 12 or 9 months	13.02 ± 1.47 (373)	12.90 ± 1.57 (177)	13.14 ± 1.29 (406)	13.10 ± 1.43 (195)					12.92 ± 1.47 (189)	12.79 ± 1.71 (97)	13.34 ± 0.97 (208)	13.32 ± 0.98 (108)				
	Study-end Δ (95% CI)	−0.21 (−0.43, 0.00)	−0.24 (−0.59, 0.11)	0.01 (−0.11, 0.14)	−0.01 (−0.22, 0.20)	0.22 (−0.02, 0.47)	0.23 (−0.18, 0.64)	0.077	0.272	−0.32 (−0.49, −0.15)	−0.31 (−0.57, −0.04)	0.06 (−0.02, 0.13)	0.14 (0.00, 0.29)	**0.38 (0.19, 0.57)**	**0.45 (0.15, 0.75)**	**<0.001**	**0.004**
**Malnutrition** [n (%) with MNA-SF ≤11]	Baseline	41 (7.7) (532)	17 (6.5) (261)	55 (10.2) (541)	28 (10.5) (267)					16 (7.7) (208)	11 (10.1) (109)	13 (5.8) (225)	9 (7.5) (120)				
	6 months	45 (10.7) (419)	27 (13.1) (206)	63 (13.0) (486)	36 (15.0) (241)	0.93 (0.57, 1.52)	0.71 (0.38, 1.32)	0.783	0.279	22 (11.6) (190)	15 (15.0) (100)	25 (12.4) (201)	12 (11.7) (103)	1.49 (0.63, 3.51)	NA	0.367	NA
	Study ends at 12 or 9 months	56 (15.0) (373)	30 (16.9) (177)	38 (9.4) (406)	23 (11.9) (195)	**0.45 (0.26, 0.78)**	0.46 (0.19, 1.11)	**0.004**	0.085	30 (15.9) (189)	17 (17.5) (97)	11 (5.3) (209)	6 (5.6) (108)	**0.39 (0.18, 0.84)**	NA	**0.016**	NA
**Instrumental Activities of Daily Living**	Baseline	7.17 ± 1.20 (533)	6.77 ± 1.44 (262)	7.39 ± 1.11 (544)	7.19 ± 1.31 (268)					7.52 ± 1.09 (210)	7.29 ± 1.35 (111)	7.39 ± 1.16 (230)	7.10 ± 1.34 (125)				
	6 months	7.08 ± 1.46 (424)	6.69 ± 1.67 (206)	7.37 ± 1.19 (488)	7.08 ± 1.39 (243)					7.22 ± 1.38 (190)	6.78 ± 1.61 (100)	7.32 ± 1.30 (200)	7.02 ± 1.52 (102)				
	6-month Δ (95% CI)	−0.12 (−0.28, 0.04)	−0.12 (−0.28, 0.04)	−0.03 (−0.20, 0.14)	−0.11 (−0.40, 0.19)	0.07 (−0.18, 0.32)	0.01 (−0.32, 0.34)	0.572	0.947	−0.30 (−0.55, −0.06)	−0.51 (−0.84, −0.19)	−0.06 (−0.22, 0.09)	−0.06 (−0.36, 0.25)	0.24 (−0.05, 0.53)	**0.46 (0.01, 0.90)**	0.108	**0.045**
	Study ends at 12 or 9 months	7.24 ± 1.26 (373)	6.82 ± 1.61 (179)	7.51 ± 1.08 (410)	7.23 ± 1.30 (197)					7.13 ± 1.52 (189)	6.66 ± 1.74 (97)	7.28 ± 1.26 (208)	7.05 ± 1.43 (107)				
	Study-end Δ (95% CI)	0.06 (−0.15, 0.27)	0.03 (−0.26, 0.32)	0.07 (−0.06, 0.20)	0.01 (−0.19, 0.21)	0.01 (−0.24, 0.25)	−0.02 (−0.37, 0.34)	0.948	0.925	−0.39 (−0.64, −0.14)	−0.62 (−0.97, −0.26)	−0.11 (−0.34, 0.12)	−0.06 (−0.29, 0.17)	0.28 (−0.06, 0.62)	**0.56 (0.13, 0.98)**	0.103	**0.010**

Y, years; CHS, Cardiovascular Health Study; MET, Metabolic equivalent of task; GDS-5, Five-item Geriatric Depression Scale; MNS-SF, Mini-Nutritional Assessment short-form; CI, Confidence interval; NA, Not available (due to unstable interaction model).

[a]Continuous variables expressed as coefficient (95% CI), binary variables as exp[coefficient] (95% CI).

[b]Generalised linear mixed model adjusted for intervention, time, intervention/time interaction, and for baseline sex, smoking habit, and Montreal Cognitive Assessment (adjusted) in the *Efficacy Study*, and for baseline education level in the *Empowerment Study*.

Chen LK, et al. Efficacy of multidomain interventions to improve physical frailty, depression and cognition: data from cluster-randomized controlled trials. *J Cachexia Sarcopenia Muscle* 2020; DOI: 10.1002/jcsm.12534.
Prof. Liang-Kung Chen, Center for Geriatrics and Gerontology, Taipei Veterans General Hospital, 201, Sec. 2, Shih-Pai Rd., Taipei 11217, Taiwan. *Email* lkchen2@vghtpe.gov.tw
