# Supplementary material for: Efficacy of multidomain interventions to improve physical frailty, depression and cognition: data from cluster‐randomized controlled trials
Source: J Cachexia Sarcopenia Muscle. 2020 Mar 5;11(3):650–62. doi: 10.1002/jcsm.12534 (PMC7296266; doi:10.1002/jcsm.12534)
Supplement: Supplementary file 2 — Table S2. Changes in participants' cognitive performance domains during the Efficacy Study and during/after the Empowerment Study [file JCSM-11-650-s007.pdf]

**Table S2** Changes in participants' cognitive performance domains during the *Efficacy Study* and during/after the *Empowerment Study*

| Outcome metrics | Assessments: Mean ± SD (*N*) | *Efficacy Study*: Intervention for 12 months |  |  |  | Assessments at baseline, 6 & 12 months |  |  |  | *Empowerment Study*: Intervention for 6 months |  |  |  | Assessments at baseline, 6 & 9 months |  |  |  |
|---|---|---|---|---|---|---|---|---|---|---|---|---|---|---|---|---|---|
|  |  | **Health education** |  | **Standard multidomain** |  | **Interaction** (95% CI)[a,b] |  | **P-value** |  | **Standard multidomain** |  | **Enhanced multidomain** |  | **Interaction** (95% CI)[a,b,c] |  | **P-value** |  |
|  |  | All | ≥75 Y | All | ≥75 Y | All | ≥75 Y | All | ≥75 Y | All | ≥75 Y | All | ≥75 Y | All | ≥75 Y | All | ≥75 Y |
| **Cognition** [$MoCA_{adj}$] | Baseline | 19.83 ± 5.50 (533) | 17.84 ± 5.56 (262) | 20.51 ± 5.83 (549) | 18.59 ± 5.86 (271) |  |  |  |  | 20.41 ± 6.51 (205) | 18.23 ± 6.65 (107) | 19.37 ± 6.36 (230) | 18.03 ± 6.38 (125) |  |  |  |  |
|  | 6 months | 19.74 ± 6.00 (420) | 17.71 ± 5.90 (208) | 20.70 ± 5.95 (481) | 18.55 ± 6.05 (240) |  |  |  |  | 21.26 ± 6.42 (190) | 18.86 ± 6.50 (100) | 20.37 ± 6.11 (201) | 18.60 ± 6.02 (103) |  |  |  |  |
|  | 6-month Δ (95% CI) | –0.29 (–1.05, 0.47) | –0.64 (–1.47, 0.19) | 0.20 (–0.46, 0.87) | 0.19 (–0.96, 1.33) | 0.49 (–0.51, 1.50) | 0.83 (–0.42, 2.08) | 0.324 | 0.183 | 0.85 (0.22, 1.48) | 0.43 (–0.44, 1.30) | 1.39 (1.05, 1.73) | 1.09 (0.53, 1.64) | 0.54 (–0.18, 1.26) | 0.66 (–0.37, 1.69) | 0.135 | 0.209 |
|  | Study ends at 12 or 9 months | 19.74 ± 6.42 (374) | 17.22 ± 6.36 (179) | 21.93 ± 5.68 (408) | 19.92 ± 5.97 (196) |  |  |  |  | 21.49 ± 6.51 (189) | 18.95 ± 6.35 (97) | 21.76 ± 6.15 (209) | 20.05 ± 6.33 (107) |  |  |  |  |
|  | Study-end Δ (95% CI) | –0.19 (–1.12, 0.74) | –0.82 (–2.24, 0.59) | 0.83 (0.05, 1.62) | 1.14 (0.17, 2.11) | 1.03 (–0.19, 2.24) | **1.96 (0.25, 3.68)** | 0.094 | **0.027** | 1.05 (0.37, 1.73) | 0.73 (–0.29, 1.76) | 2.34 (2.02, 2.66) | 1.97 (1.54, 2.41) | **1.29 (0.54, 2.03)** | **1.24 (0.13, 2.35)** | **<0.001** | **0.029** |
| Visuospatial Executive | Baseline | 2.49 ± 1.72 (533) | 1.95 ± 1.63 (262) | 2.71 ± 1.74 (549) | 2.32 ± 1.72 (271) |  |  |  |  | 2.84 ± 1.70 (210) | 2.41 ± 1.65 (111) | 2.66 ± 1.56 (230) | 2.38 ± 1.60 (125) |  |  |  |  |
|  | 6 months | 2.51 ± 1.76 (421) | 2.00 ± 1.68 (208) | 2.88 ± 1.69 (484) | 2.48 ± 1.61 (240) |  |  |  |  | 3.10 ± 1.57 (190) | 2.73 ± 1.54 (100) | 2.66 ± 1.61 (201) | 2.31 ± 1.63 (103) |  |  |  |  |
|  | 6-month Δ (95% CI) | –0.02 (–0.21, 0.17) | –0.04 (–0.26, 0.17) | 0.16 (–0.05, 0.37) | 0.19 (–0.88, 1.26) | 0.18 (–0.10, 0.46) | 0.23 (0.12, 0.58) | 0.200 | 0.195 | 0.28 (–0.09, 0.66) | 0.29 (–0.24, 0.81) | 0.11 (–0.06, 0.27) | 0.06 (–0.19, 0.31) | –0.18 (–0.58, 0.23) | –0.23 (–0.80, 0.34) | 0.373 | 0.424 |
|  | Study ends at 12 or 9 months | 2.55 ± 1.78 (374) | 1.99 ± 1.68 (179) | 3.00 ± 1.71 (409) | 2.63 ± 1.70 (197) |  |  |  |  | 3.12 ± 1.59 (189) | 2.68 ± 1.49 (97) | 2.91 ± 1.58 (209) | 2.62 ± 1.66 (107) |  |  |  |  |
|  | Study-end Δ (95% CI) | 0.07 (–0.34, 0.48) | 0.02 (–0.44, 0.49) | 0.11 (–0.24, 0.46) | 0.22 (–0.09, 0.53) | 0.04 (–0.49, 0.58) | 0.20 (–0.36, 0.76) | 0.877 | 0.476 | 0.28 (0.08, 0.49) | 0.28 (0.05, 0.51) | 0.23 (0.08, 0.39) | 0.22 (0.02, 0.42) | –0.05 (–0.31, 0.20) | –0.06 (–0.36, 0.24) | 0.694 | 0.694 |
| Naming[c] | Baseline | 2.32 ± 0.94 (533) | 2.13 ± 1.01 (262) | 2.39 ± 0.92 (549) | 2.20 ± 1.01 (271) |  |  |  |  | 2.16 ± 1.03 (205) | 1.91 ± 1.15 (107) | 1.91 ± 1.12 (230) | 1.73 ± 1.13 (125) |  |  |  |  |
|  | 6 months | 2.34 ± 0.95 (420) | 2.18 ± 1.01 (208) | 2.36 ± 0.94 (483) | 2.13 ± 1.02 (240) |  |  |  |  | 2.15 ± 1.03 (190) | 1.90 ± 1.06 (100) | 2.00 ± 1.04 (201) | 1.83 ± 1.10 (103) |  |  |  |  |
|  | 6-month Δ (95% CI) | 0.00 (–0.10, 0.09) | –0.01 (–0.22, 0.19) | –0.04 (–0.12, 0.05) | –0.05 (0.98, 0.88) | –0.03 (–0.16, 0.09) | –0.04 (–0.23, 0.16) | 0.615 | 0.713 | –0.02 (–0.18, 0.14) | –0.03 (–0.26, 0.21) | 0.14 (0.02, 0.26) | 0.18 (0.05, 0.31) | 0.16[c] (–0.04, 0.36) | 0.21[c] (–0.07, 0.48) | 0.108 | 0.130 |
|  | Study ends at 12 or 9 months | 2.34 ± 1.00 (374) | 2.19 ± 1.06 (179) | 2.56 ± 0.80 (408) | 2.37 ± 0.95 (196) |  |  |  |  | 2.18 ± 1.00 (189) | 1.88 ± 1.03 (97) | 2.15 ± 1.00 (209) | 2.06 ± 1.04 (107) |  |  |  |  |

| | | | | | | | | | | | | | | | | | |
|---|---|---|---|---|---|---|---|---|---|---|---|---|---|---|---|---|---|
| | Study-end Δ (95% CI) | 0.01 (−0.10, 0.13) | 0.07 (−0.10, 0.23) | 0.11 (0.02, 0.19) | 0.16 (0.02, 0.30) | 0.09 (−0.05, 0.23) | 0.09 (−0.12, 0.31) | 0.193 | 0.396 | 0.04 (−0.13, 0.20) | 0.01 (−0.25, 0.28) | 0.20 (0.08, 0.33) | 0.29 (0.19, 0.40) | 0.17[c] (−0.04, 0.38) | 0.28[c] (0.00, 0.56) | 0.119 | 0.054 |
| Concentration[c] | Baseline | 4.39 ± 1.55 (533) | 3.95 ± 1.60 (262) | 4.38 ± 1.62 (549) | 4.01 ± 1.72 (271) | | | | | 4.57 ± 1.62 (210) | 4.30 ± 1.73 (111) | 4.22 ± 1.68 (230) | 3.90 ± 1.80 (125) | | | | |
| | 6 months | 4.38 ± 1.54 (420) | 4.13 ± 1.59 (208) | 4.56 ± 1.49 (486) | 4.22 ± 1.64 (242) | | | | | 4.65 ± 1.62 (190) | 4.17 ± 1.78 (100) | 4.33 ± 1.64 (201) | 3.95 ± 1.66 (103) | | | | |
| | 6-month Δ (95% CI) | −0.06 (−0.15, 0.03) | 0.05 (0.48, 0.58) | 0.17 (0.00, 0.34) | 0.24 (−0.55, 1.03) | **0.23 (0.04, 0.42)** | 0.19 (−0.09, 0.46) | **0.019** | 0.182 | 0.07 (−0.14, 0.27) | −0.18 (−0.53, 0.18) | 0.18 (−0.06, 0.42) | 0.16 (−0.05, 0.38) | 0.11[c] (−0.21, 0.43) | 0.34[c] (−0.08, 0.76) | 0.489 | 0.108 |
| | Study ends at 12 or 9 months | 4.21 ± 1.65 (374) | 3.77 ± 1.82 (179) | 4.74 ± 1.43 (408) | 4.45 ± 1.52 (196) | | | | | 4.51 ± 1.65 (189) | 4.08 ± 1.72 (97) | 4.44 ± 1.72 (209) | 4.09 ± 1.84 (107) | | | | |
| | Study-end Δ (95% CI) | −0.19 (−0.31, −0.06) | −0.20 (−0.44, 0.03) | 0.27 (0.06, 0.48) | 0.44 (0.23, 0.65) | **0.46 (0.22, 0.70)** | **0.64 (0.32, 0.95)** | **<0.001** | **<0.001** | −0.05 (−0.33, 0.23) | −0.21 (−0.64, 0.23) | 0.17 (−0.01, 0.35) | 0.16 (−0.12, 0.43) | 0.22[c] (−0.11, 0.55) | 0.36[c] (−0.14, 0.87) | 0.194 | 0.152 |
| Language | Baseline | 1.55 ± 1.02 (533) | 1.33 ± 1.00 (262) | 1.78 ± 0.99 (549) | 1.55 ± 1.01 (271) | | | | | 1.74 ± 1.02 (210) | 1.49 ± 0.96 (111) | 1.71 ± 0.94 (230) | 1.48 ± 0.90 (125) | | | | |
| | 6 months | 1.44 ± 1.07 (420) | 1.21 ± 1.05 (208) | 1.78 ± 1.01 (487) | 1.45 ± 1.01 (242) | | | | | 1.85 ± 0.96 (190) | 1.47 ± 0.92 (100) | 1.83 ± 0.94 (201) | 1.59 ± 0.97 (103) | | | | |
| | 6-month Δ (95% CI) | −0.12 (−0.25, 0.02) | −0.16 (−0.54, 0.22) | −0.03 (−0.15, 0.10) | −0.10 (−0.26, 0.07) | 0.09 (−0.10, 0.28) | 0.07 (−0.20, 0.33) | 0.337 | 0.623 | 0.10 (−0.07, 0.27) | −0.03 (−0.26, 0.20) | 0.14 (−0.10, 0.37) | 0.14 (−0.20, 0.48) | 0.04 (−0.26, 0.33) | 0.17 (−0.24, 0.58) | 0.808 | 0.418 |
| | Study ends at 12 or 9 months | 1.56 ± 1.05 (374) | 1.29 ± 1.06 (179) | 1.91 ± 1.00 (409) | 1.51 ± 1.03 (197) | | | | | 1.85 ± 0.98 (189) | 1.61 ± 1.01 (97) | 1.95± 0.95 (209) | 1.78 ± 0.92 (107) | | | | |
| | Study-end Δ (95% CI) | −0.05 (−0.27, 0.17) | −0.09 (−0.32, 0.14) | 0.07 (−0.07, 0.20) | −0.05 (−0.28, 0.17) | 0.12 (−0.14, 0.37) | 0.04 (−0.28, 0.36) | 0.351 | 0.998 | 0.10 (−0.07, 0.28) | 0.12 (−0.06, 0.30) | 0.23 (−0.02, 0.47) | 0.29 (0.03, 0.55) | 0.12 (−0.17, 0.42) | 0.17 (−0.14, 0.49) | 0.412 | 0.286 |
| Abstract Thinking[c] | Baseline | 0.61 ± 0.76 (533) | 0.49 ± 0.71 (262) | 0.68 ± 0.78 (549) | 0.57 ± 0.73 (271) | | | | | 0.74 ± 0.83 (210) | 0.57 ± 0.77 (111) | 0.55 ± 0.74 (230) | 0.51 ± 0.71 (125) | | | | |
| | 6 months | 0.56 ± 0.78 (420) | 0.40 ± 0.67 (208) | 0.63 ± 0.77 (487) | 0.49 ± 0.71 (242) | | | | | 0.66 ± 0.82 (190) | 0.48 ± 0.72 (100) | 0.50 ± 0.72 (201) | 0.36 ± 0.64 (103) | | | | |
| | 6-month Δ (95% CI) | −0.07 (−0.18, 0.03) | −0.13 (−0.40, 0.15) | −0.04 (−0.18, 0.10) | −0.05 (−0.37, 0.27) | 0.04 (−0.14, 0.21) | 0.08 (−0.09, 0.24) | 0.691 | 0.367 | −0.07 (−0.25, 0.12) | −0.10 (−0.28, 0.08) | 0.00 (−0.08, 0.09) | −0.10 (−0.19, −0.01) | 0.07[c] (−0.13, 0.27) | 0.00[c] (−0.20, 0.20) | 0.473 | 0.971 |
| | Study ends at 12 or 9 months | 0.55 ± 0.77 (374) | 0.35 ± 0.63 (179) | 0.70 ± 0.82 (409) | 0.53 ± 0.78 (197) | | | | | 0.66 ± 0.81 (189) | 0.44 ± 0.75 (97) | 0.59 ± 0.78 (209) | 0.52 ± 0.76 (107) | | | | |
| | Study-end Δ (95% CI) | −0.06 (−0.14, 0.02) | −0.15 (−0.26, −0.03) | −0.03 (−0.16, 0.11) | −0.07 (−0.18, 0.05) | 0.04 (−0.13, 0.20) | 0.08 (−0.08, 0.24) | 0.669 | 0.332 | −0.07 (−0.18, 0.05) | −0.10 (−0.26, 0.06) | 0.03 (−0.02, 0.07) | 0.00 (−0.09, 0.10) | 0.09[c] (−0.03, 0.21) | 0.11[c] (−0.08, 0.29) | 0.137 | 0.256 |

| | | | | | | | | | | | | | | | | | |
|---|---|---|---|---|---|---|---|---|---|---|---|---|---|---|---|---|---|
| Delayed Recall | Baseline | 2.23 ± 1.64 (533) | 1.86 ± 1.70 (262) | 2.29 ± 1.72 (549) | 1.81 ± 1.74 (271) | | | | | 2.40 ± 1.89 (210) | 1.86 ± 1.92 (111) | 2.21 ± 1.74 (230) | 2.05 ± 1.72 (125) | | | | |
| | 6 months | 2.23 ± 1.82 (420) | 1.74 ± 1.79 (208) | 2.31 ± 1.76 (488) | 1.88 ± 1.76 (242) | | | | | 2.66 ± 1.78 (190) | 2.11 ± 1.84 (100) | 2.82 ± 1.58 (201) | 2.47 ± 1.66 (103) | | | | |
| | 6-month Δ (95% CI) | –0.03 (-0.43, 0.37) | –0.20 (-0.48, 0.08) | –0.01 (-0.24, 0.22) | 0.06 (-0.79, 0.92) | 0.02 (-0.44, 0.49) | 0.26 (-0.27, 0.79) | 0.927 | 0.326 | 0.27 (-0.06, 0.61) | 0.23 (-0.11, 0.58) | 0.63 (0.41, 0.85) | 0.46 (0.19, 0.74) | 0.36 (-0.04, 0.76) | 0.23 (-0.21, 0.67) | 0.080 | 0.306 |
| | Study ends at 12 or 9 months | 2.33 ± 1.83 (374) | 1.63 ± 1.71 (179) | 2.71 ± 1.82 (409) | 2.30 ± 1.89 (197) | | | | | 2.96 ± 1.79 (189) | 2.27 ± 1.76 (97) | 3.41 ± 1.62 (209) | 2.94 ± 1.76 (107) | | | | |
| | Study-end Δ (95% CI) | 0.07 (-0.27, 0.41) | –0.28 (-0.70, 0.13) | 0.33 (-0.04, 0.70) | 0.47 (0.03, 0.90) | 0.26 (-0.24, 0.76) | **0.75 (0.15, 1.35)** | 0.302 | **0.015** | 0.56 (0.16, 0.96) | 0.42 (-0.03, 0.86) | 1.19 (1.03, 1.35) | 0.89 (0.67, 1.11) | **0.63 (0.20, 1.06)** | 0.47 (-0.02, 0.97) | **0.004** | 0.061 |
| Orientation | Baseline | 5.31 ± 1.10 (533) | 5.16 ± 1.19 (262) | 5.35 ± 1.11 (549) | 5.15 ± 1.22 (271) | | | | | 5.08 ± 1.32 (210) | 4.76 ± 1.48 (111) | 5.14 ± 1.23 (230) | 5.02 ± 1.23 (125) | | | | |
| | 6 months | 5.32 ± 1.09 (420) | 5.09 ± 1.21 (208) | 5.39 ± 1.00 (488) | 5.11 ± 1.15 (242) | | | | | 5.32 ± 1.12 (190) | 5.03 ± 1.34 (100) | 5.26 ± 1.02 (201) | 5.12 ± 1.07 (103) | | | | |
| | 6-month Δ (95% CI) | 0.00 (-0.12, 0.12) | –0.13 (-1.13, 0.87) | 0.04 (-0.07, 0.16) | –0.01 (-0.90, 0.88) | 0.04 (-0.12, 0.21) | 0.12 (-0.11, 0.35) | 0.622 | 0.316 | 0.25 (0.13, 0.37) | 0.26 (-0.02, 0.53) | 0.17 (-0.03, 0.37) | 0.17 (-0.15, 0.49) | –0.08 (-0.31, 0.16) | –0.09 (-0.51, 0.33) | 0.522 | 0.678 |
| | Study ends at 12 or 9 months | 5.30 ± 1.05 (374) | 5.06 ± 1.15 (179) | 5.44 ± 0.98 (409) | 5.20 ± 1.11 (197) | | | | | 5.35 ± 1.06 (189) | 5.03 ± 1.25 (97) | 5.38 ± 1.06 (209) | 5.09 ± 1.26 (107) | | | | |
| | Study-end Δ (95% CI) | –0.04 (-0.16, 0.09) | –0.14 (-0.29, 0.01) | 0.00 (-0.09, 0.10) | 0.01 (-0.15, 0.16) | 0.04 (-0.12, 0.19) | 0.15 (-0.07, 0.36) | 0.633 | 0.177 | 0.28 (0.18, 0.38) | 0.29 (0.09, 0.49) | 0.23 (0.14, 0.32) | 0.07 (-0.08, 0.22) | –0.05 (-0.19, 0.08) | –0.23 (-0.47, 0.02) | 0.449 | 0.072 |

Y, years; $MoCA_{adj}$, Montreal Cognitive Assessment (adjusted); CI, Confidence interval.

[a]Continuous variables expressed as coefficient (95% CI), binary variables as exp[coefficient] (95% CI).

[b]Linear mixed model adjusted for intervention, time, intervention/time interaction, and for baseline sex, smoking habit, and $MoCA_{adj}$ in the *Efficacy Study*, and for baseline education level in the *Empowerment Study*.

[c]Analysis of intervention effect adjusted for between-group differences in baseline naming, concentration, and abstract thinking in the *Empowerment Study*.

Chen LK, et al. Efficacy of multidomain interventions to improve physical frailty, depression and cognition: data from cluster-randomized controlled trials. *J Cachexia Sarcopenia Muscle* 2020; DOI: 10.1002/jcsm.12534.
Prof. Liang-Kung Chen, Center for Geriatrics and Gerontology, Taipei Veterans General Hospital, 201, Sec. 2, Shih-Pai Rd., Taipei 11217, Taiwan. *Email* lkchen2@vghtpe.gov.tw
