# Supplementary material for: Efficacy of multidomain interventions to improve physical frailty, depression and cognition: data from cluster‐randomized controlled trials
Source: J Cachexia Sarcopenia Muscle. 2020 Mar 5;11(3):650–62. doi: 10.1002/jcsm.12534 (PMC7296266; doi:10.1002/jcsm.12534)
Supplement: Supplementary file 3 — Table S3. Combined effect of interventions on cognitive, physical, and function domains at 6 months [file JCSM-11-650-s008.pdf]

Table S3a Combined effect of interventions on cognitive, physical, and function domains at 6 months

Metric	*Efficacy Study*: (Direct comparison at 6-month follow-up)			*Empowerment Study*: (Direct comparison at 6-month follow-up)			**Efficacy + Empowerment** (Indirect comparison)
	Mean change[a] ± SD		Cohen's *d*[b] (pooled SD)	Mean change[a] ± SD		Cohen's *d*[b] (pooled SD)	Cohen's *d*
	Health education	Standard multidomain	(Multidomain – Health education)	Standard multidomain	Enhanced multidomain	(Enhanced – Standard)	(Combined effect)
CHS Frailty score	–0.01 (1.00)	–0.14 (0.97)	0.13 (0.98)[c]	–0.01 (0.95)	–0.22 (0.95)	0.22 (0.95)[c]	0.35
Gait speed	0.03 (0.34)	0.07 (0.31)	0.13 (0.32)	–0.01 (0.34)	0.05 (0.31)	0.19 (0.32)	0.32
Physical activity	0.23 (17.2)	2.84 (18.6)	0.15 (17.9)	0.02 (17.7)	4.68 (22.0)	0.23 (20.1)	0.38
$MoCA_{adj}$	–0.29 (5.73)	0.20 (5.89)	0.08 (5.81)	0.85 (6.47)	1.39 (6.25)	0.09 (6.35)	0.17
Concentration	–0.06 (1.55)	0.17 (1.56)	0.15 (1.56)	0.07 (1.62)	0.18 (1.66)	0.05 (1.64)	0.15
Delayed recall	–0.03 (1.72)	–0.01 (1.74)	0.01 (1.73)	0.27 (1.84)	0.63 (1.67)	0.21 (1.75)	0.22
IADL	–0.12 (1.32)	–0.03 (1.15)	0.07 (1.24)	–0.30 (1.23)	–0.06 (1.23)	0.20 (1.23)	0.27
GDS-5	–0.01 (0.83)	–0.02 (0.84)	0.01 (0.84)[c]	–0.01 (0.83)	0.07 (0.83)	–0.10 (0.83)[c]	–0.09
MNA-SF	–0.04 (1.19)	–0.05 (1.28)	-0.01 (1.24)	–0.14 (1.18)	–0.26 (1.13)	–0.10 (1.15)	-0.11

Table S3b Combined effect of interventions on cognitive, physical, and function domains at 12 or 9 months

Metric	*Efficacy Study* (Direct comparison at 12-month follow-up)			*Empowerment Study* (Direct comparison at 9-month follow-up)			**Efficacy + Empowerment** (Indirect comparison)
	Mean change[a] ± SD		Cohen's *d*[b] (pooled SD)	Mean change[a] ± SD		Cohen's *d*[b] (pooled SD)	Cohen's *d*
	Health education	Standard multidomain	(Multidomain – Health education)	Standard multidomain	Enhanced multidomain	(Enhanced – Standard)	(Combined effect)
CHS Frailty score	–0.02 (1.00)	–0.14 (0.96)	0.12 (0.98)[c]	–0.13 (0.98)	–0.18 (0.97)	0.05 (0.97)[c]	0.17
Gait speed	0.06 (0.34)	0.08 (0.31)	0.06 (0.33)	0.02 (0.35)	0.00 (0.31)	–0.07 (0.32)	-0.01
Physical activity	1.48 (19.6)	0.64 (15.5)	–0.05 (17.6)	1.05 (18.4)	1.90 (23.7)	0.04 (20.0)	-0.01
$MoCA_{adj}$	–0.19 (5.90)	0.83 (5.77)	0.17 (5.83)	1.05 (6.51)	2.34 (6.26)	0.20 (6.38)	0.37
Concentration	–0.19 (1.60)	0.27 (1.54)	0.29 (1.57)	–0.05 (1.63)	0.17 (1.70)	0.13 (1.67)	0.15
Delayed recall	0.07 (1.72)	0.33 (1.76)	0.15 (1.74)	0.56 (1.84)	1.19 (1.68)	0.36 (1.76)	0.51
IADL	0.06 (1.23)	0.07 (1.10)	0.01 (1.16)	-0.39 (1.31)	-0.11 (1.21)	0.22 (1.26)	0.23
GDS-5	0.04 (0.86)	-0.07 (0.87)	0.13 (0.87)[c]	0.16 (0.87)	0.07 (0.91)	0.10 (0.89)[c]	0.23
MNA-SF	-0.21(1.29)	0.01 (1.27)	0.17 (1.28)	-0.32 (1.26)	0.06 (1.01)	0.31 (1.13)	0.42

SD, Standard deviation; $MoCA_{adj}$, Montreal Cognitive Assessment (adjusted); CHS, Cardiovascular Health Study; IADL, Instrumental activities of daily living; GDS-5, Five-item Geriatric Depression Scale; MNA-SF Mini-Nutritional Assessment short-form.

[a]Mean changes were derived from the generalised linear mixed model.

[b]Cohen's *d* was calculated by [(mean change of Standard multidomain – mean change of Health promotion), or (mean change of Enhanced multidomain – mean change of Standard multidomain)]/pooled SD.

[c]Since CHS frailty score and GDS-5 were negative indicators, Cohen's *d* was calculated thus: −[(Standard multidomain − Health promotion)/pooled SD, or –[(Enhanced multidomain – Standard multidomain)/pooled SD].

Chen LK, et al. Efficacy of multidomain interventions to improve physical frailty, depression and cognition: data from cluster-randomized controlled trials. *J Cachexia Sarcopenia Muscle* 2020; DOI: 10.1002/jcsm.12534.
Prof. Liang-Kung Chen, Center for Geriatrics and Gerontology, Taipei Veterans General Hospital, 201, Sec. 2, Shih-Pai Rd., Taipei 11217, Taiwan. *Email* lkchen2@vghtpe.gov.tw
