# Supplementary material for: Efficacy of multidomain interventions to improve physical frailty, depression and cognition: data from cluster‐randomized controlled trials
Source: J Cachexia Sarcopenia Muscle. 2020 Mar 5;11(3):650–62. doi: 10.1002/jcsm.12534 (PMC7296266; doi:10.1002/jcsm.12534)
Supplement: Supplementary file 4 — Appendix S1. Efficacy and Empowerment Studies: standard and enhanced multidomain intervention protocols [file JCSM-11-650-s001.pdf]

Appendix S1 *Efficacy and Empowerment Studies*: standard and enhanced multidomain intervention protocols

Standard multidomain interventions and schedule

	**Physical fitness** (~45 min)	**Cognitive training** (~60 min)[a]	**Nutritional advice** (~15 min)	**Disease education** (~30–60 min)[a]
**Month**	**Warm-up** (5 mins)<br>• At instructor's discretion<br>**Strength and balance** (30 mins)<br>• At instructor's discretion (eg, resistance band/weight, exercise ball, free-weight postures; one-legged stand, tandem walk)<br>**Stretching and flexibility** (10 mins)<br>• At instructor's discretion	**Reasoning**<br>• Logic puzzles (eg, Tangram, Suduko)<br>• Pattern recognition (eg, find the correct start/stop station in a public transport timetable, and the next possible alternative)<br>**Memory**<br>• Mnemonic strategies for remembering verbal material (eg, memorize a shopping list)	**Nutritional literacy**<br>• General information on healthy nutrition and diet based on the *Dietary Guideline of Taiwan*,[1] at instructor's discretion<br>• Balanced dietary choices including macronutrients and micronutrients<br>• Maintaining adequate protein intake	**Chronic disease prevention class**<br>• Successful aging<br>• Introduction to dementia<br>• Management of metabolic syndrome and other cardiovascular risk factors<br>• Osteoporosis<br>• Sarcopenia
**1:**	Four sessions (45 min/week)	Four sessions (1 hr/week)	Four sessions (15 min/week)	Three or four/16 sessions, every 3 to 4 months (1.5–4 hours/year)[a]
**2:**	Two sessions (45 min/fortnight)	Two sessions (1 hr/fortnight)	Two sessions (15 min/fortnight)	
**3–12:**	Ten sessions (45 min/month)	Ten sessions (1 hr/month)	Ten sessions (15 min/month)	

[a]Every 3 to 4 months, some physical fitness, cognitive training, and/or nutritional advice activities were curtailed to make time for a visiting doctor to give a class on preventing/managing chronic disease. One or two such sessions were held during the 6-month *Empowerment Study* intervention.

Efficacy Study

The Taiwan Health Promotion Intervention Study for Elders used a multidomain intervention similar to that used in the French 'Multidomain Alzheimer Preventive Trial (MAPT),[2] which evaluated interventions that were widely available to older adults, easy to organise in community settings, and well accepted by participants.

The physical exercise, cognitive training, and nutritional interventions were designed to foster healthy lifestyle behaviours, and to motivate participants to practice themselves at home. Training sessions during the first and second months were more frequent, to ingrain healthy lifestyle behaviours, followed by regular maintenance sessions to consolidate gains. Clusters of 20–50 people were divided into smaller groups of 5–8 participants per session to promote effective delivery. Every 3 to 4 months, an invited doctor gave an additional 30–60 min disease education class.

Physical fitness

Physical exercise classes were conducted by professional fitness coaches, trained part-time staff, or physical therapists, and entailed activities focused on improving strength, balance, and flexibility. The protocol did not specify particular exercises, equipment or timings, but the instructors principally used simple and widely available commercial products such as elastic-resistance bands, water bottles (as weights), exercise balls, chairs, or other aids. Besides, participants were actively encouraged to follow the Taiwan Health Promotion Administration (HPA) recommendation of undertaking at least 150 min/week of moderate to vigorous physical activities;[3] as walking is the simplest, the HPA recommends walking 30 min/day. However, participants could choose other activities to do in their own time, such as aerobic exercise or folk dancing.

Cognitive training

The goal of cognitive training is to enhance skills that will help elderly people to tackle challenges arising in their daily lives; therefore, cognitive training focused on reasoning and memory domains. Sessions were conducted by an occupational therapist or a trained instructor. There was no set duration or order of activities, but each group had to receive both reasoning and memory training in each session. Reasoning exercises included logic puzzles and recognising patterns in word/number lists to determine the next item; a practical example was finding the correct start/stop station in a public transport timetable and identifying the next possible alternative. Memory training include teaching mnemonic skills of organization, visual imagination and association to memorize story texts or word lists, such as remembering a shopping list.

Nutritional advice

Healthy nutrition education based on Taiwan HPA dietary guidelines[1] was led by a dietician at most sites, but could also be provided by an instructor trained to deliver the other multidomain intervention components. Besides giving general advice to improve participants' nutritional literacy, instructors were specifically tasked to cover the importance of choosing a balanced diet across macro- and micronutrient categories, and maintaining an adequate protein intake.

Empowerment Study

The multidomain intervention program in the *Empowerment Study* replicated the format and schedule of the first 6 months (10 sessions) of the *Efficacy Study* but was enhanced in several ways to try and consolidate healthy lifestyle changes among the participants. Following feedback from *Efficacy Study* participants and a needs-assessment survey in local communities, health promotion experts revised and simplified the standard multidomain protocol to produce new training materials. The research team pretested the new protocol in groups of people who did not participate in the ensuing study, then made further revisions to finalize the intervention program.

In addition to the standard interventions, each participant was given a pedometer and a sheet to record their daily number of steps; pedometer data were returned at the next class and participants were encouraged to increase the number of steps by 10%/week, or to take 10,000 steps/day. Post-curriculum learning sheets for cognitive training and nutrition classes were designed to enable participants to put what they had learned into practice, and research staff gave everyone feedback on their after-class 'homework' at the next training session.

At the group level, an elected community-leader was made responsible for motivating and monitoring their members' participation. Competitions with small prizes (valued ~USD 2–3) for the winners were also held. Intervention activities and participant experience sharing were photographed and videoed, and these documentary materials were sent to the participating communities and individuals to encourage them to maintain a healthy lifestyle after the study ended.

References

1. Health Promotion Administration, Ministry of Health and Welfare, Taiwan. Dietary Guideline of Taiwan, 2016. https://www.hpa.gov.tw/Pages/ashx/File.ashx?FilePath=~/File/Attach/7365/File_6870.pdf
2. Vellas B, et al. MAPT Study: A multidomain approach for preventing Alzheimer's disease: design and baseline data. *J Prev Alzheimers Dis* 2014;**1**:13–22.
3. Health Promotion Administration, Ministry of Health and Welfare, Taiwan. Health Promotion Administration, Annual Report 2015. https://health99.hpa.gov.tw/media/public/pdf/21791.pdf

Chen LK, et al. Efficacy of multidomain interventions to improve physical frailty, depression and cognition: data from cluster-randomized controlled trials. *J Cachexia Sarcopenia Muscle* 2020; DOI: 10.1002/jcsm.12534.
Prof. Liang-Kung Chen, Center for Geriatrics and Gerontology, Taipei Veterans General Hospital, 201, Sec. 2, Shih-Pai Rd., Taipei 11217, Taiwan. *Email* lkchen2@vghtpe.gov.tw
