# Supplementary material for: Efficacy of multidomain interventions to improve physical frailty, depression and cognition: data from cluster‐randomized controlled trials
Source: J Cachexia Sarcopenia Muscle. 2020 Mar 5;11(3):650–62. doi: 10.1002/jcsm.12534 (PMC7296266; doi:10.1002/jcsm.12534)
Supplement: Supplementary file 5 — Figure S1. Efficacy and Empowerment Studies intervention and assessment schemes [file JCSM-11-650-s002.pdf]

Figure S1 *Efficacy* and *Empowerment Studies* intervention and assessment schemes

Efficacy Study

Control:
- Periodic telephone health promotion (~3-monthly)

Standard multidomain intervention:
- Physical exercise (45 min), cognitive training (60 min), diet advice (15 min)
- 3–4 disease education classes (30–60 min)

Empowerment Study

Control:
- Standard multidomain intervention

Enhanced multidomain intervention:
- Revised *Efficacy Study* teaching materials
- Physical exercise (45 min), cognitive training (60 min), diet advice (15 min)
- 1–2 disease education classes (30–60 min)

PLUS:
- Post-curriculum learning sheets
- Goal-setting & monitoring (eg, pedometer)
- Community-leader involvement
- Individual encouragement & motivation

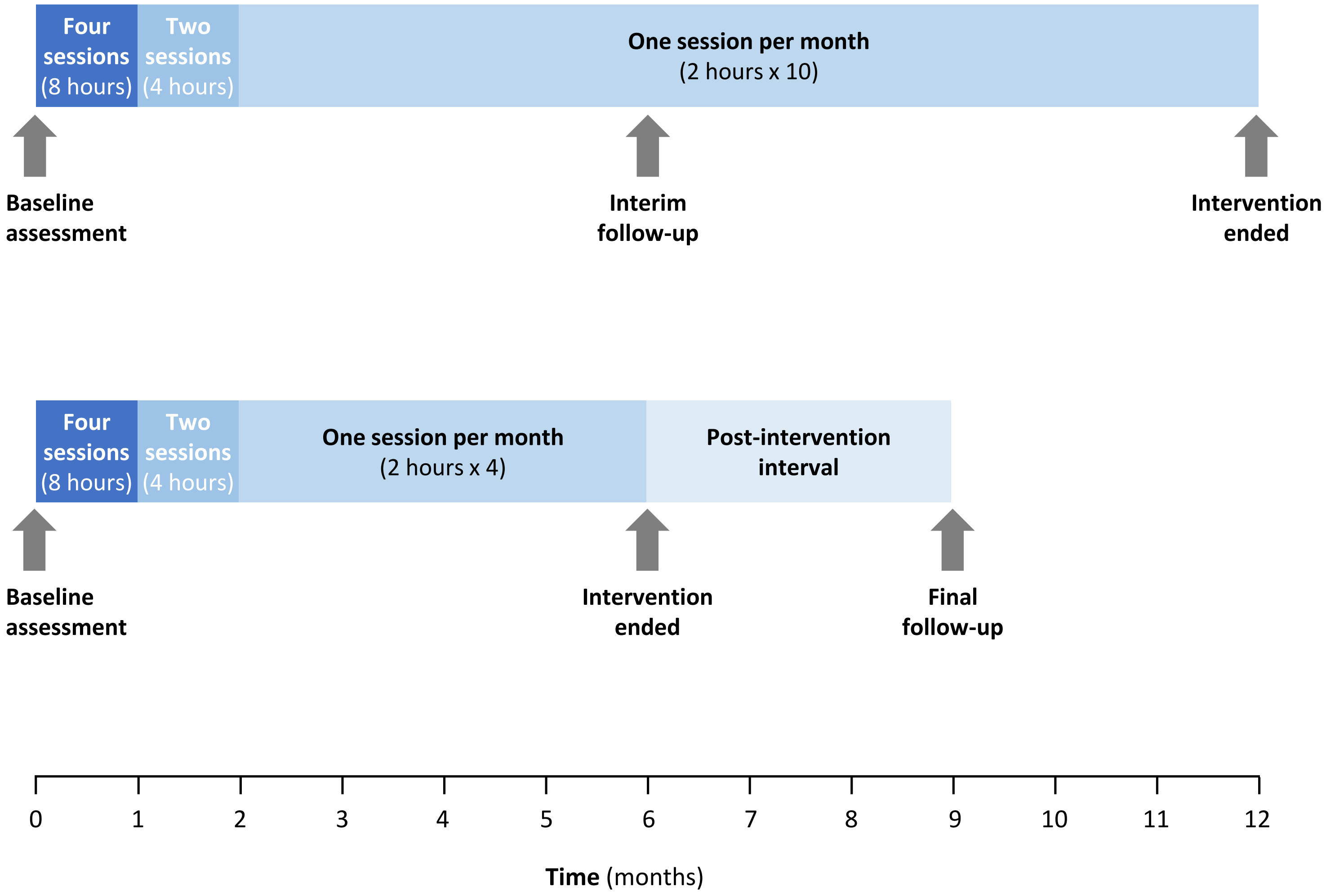

Chen LK, et al. Efficacy of multidomain interventions to improve physical frailty, depression and cognition: data from cluster-randomized controlled trials. *J Cachexia Sarcopenia Muscle* 2020; DOI: 10.1002/jcsm.12534.
Prof. Liang-Kung Chen, Center for Geriatrics and Gerontology, Taipei Veterans General Hospital, 201, Sec. 2, Shih-Pai Rd., Taipei 11217, Taiwan. Email lkchen2@vghtpe.gov.tw
