# Supplementary material for: Efficacy of multidomain interventions to improve physical frailty, depression and cognition: data from cluster‐randomized controlled trials
Source: J Cachexia Sarcopenia Muscle. 2020 Mar 5;11(3):650–62. doi: 10.1002/jcsm.12534 (PMC7296266; doi:10.1002/jcsm.12534)
Supplement: Supplementary file 6 — Figure S2. Mean changes from baseline performance among participants ≥75 years old [file JCSM-11-650-s003.pdf]

**Figure S2** Mean changes from baseline performance among ≥75-years-olds: **A)** Physical domains; **B)** Functional domains; **C)** Cognitive domains

* $P < 0.05$; ** $P < 0.01$; *** $P < 0.001$; Vertical bars indicate standard error.

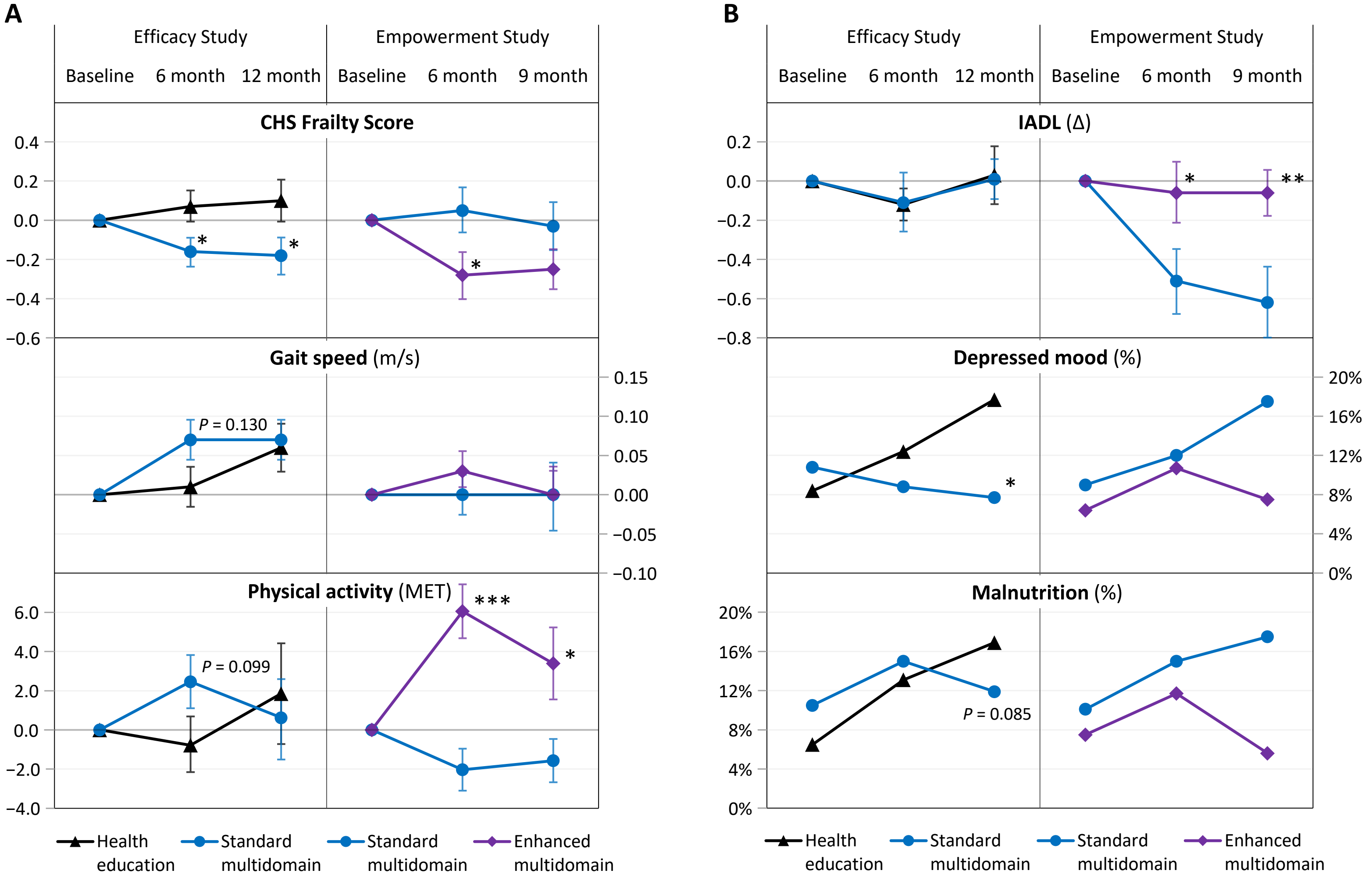


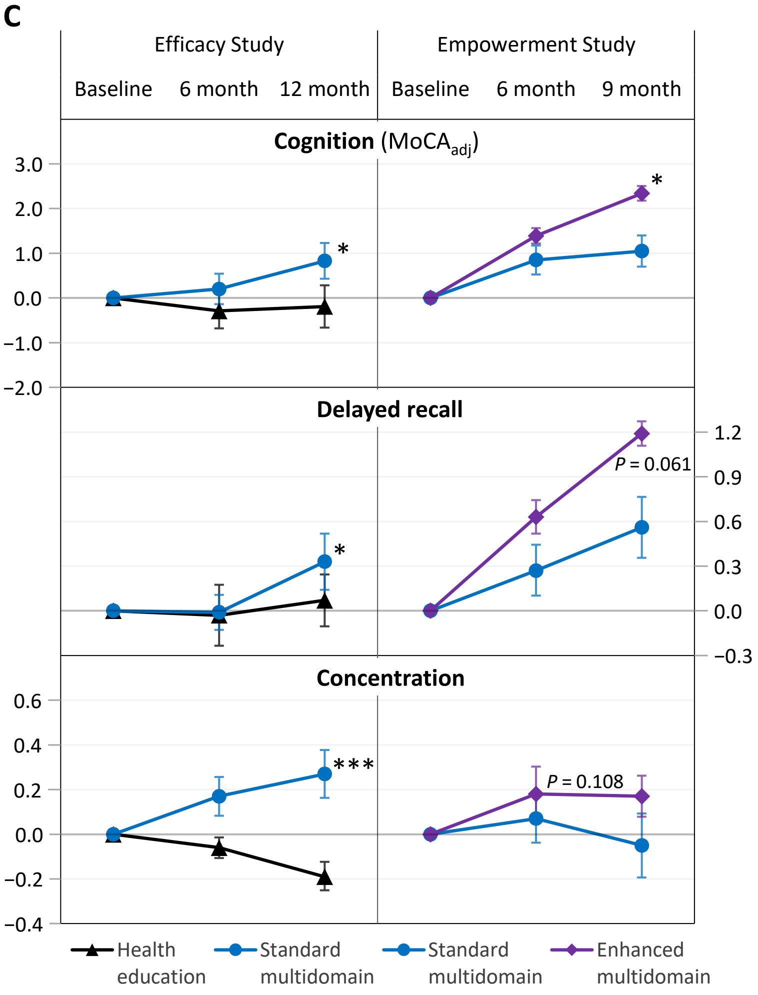



Chen LK, et al. Efficacy of multidomain interventions to improve physical frailty, depression and cognition: data from cluster-randomized controlled trials.
*J Cachexia Sarcopenia Muscle* 2020;
DOI: 10.1002/jcsm.12534.


Prof. Liang-Kung Chen, Center for Geriatrics and Gerontology, Taipei Veterans General Hospital, 201, Sec. 2, Shih-Pai Rd., Taipei 11217, Taiwan.
*Email* lkchen2@vghtpe.gov.tw
