# Supplementary material for: Efficacy of multidomain interventions to improve physical frailty, depression and cognition: data from cluster‐randomized controlled trials
Source: J Cachexia Sarcopenia Muscle. 2020 Mar 5;11(3):650–62. doi: 10.1002/jcsm.12534 (PMC7296266; doi:10.1002/jcsm.12534)
Supplement: Supplementary file 7 — Figure S3. Intervention effects on physical and functional performance among participants ≥75 years old [file JCSM-11-650-s004.pdf]

Figure S3A Efficacy Study intervention effects on physical and functional performance among participants ≥75 years old

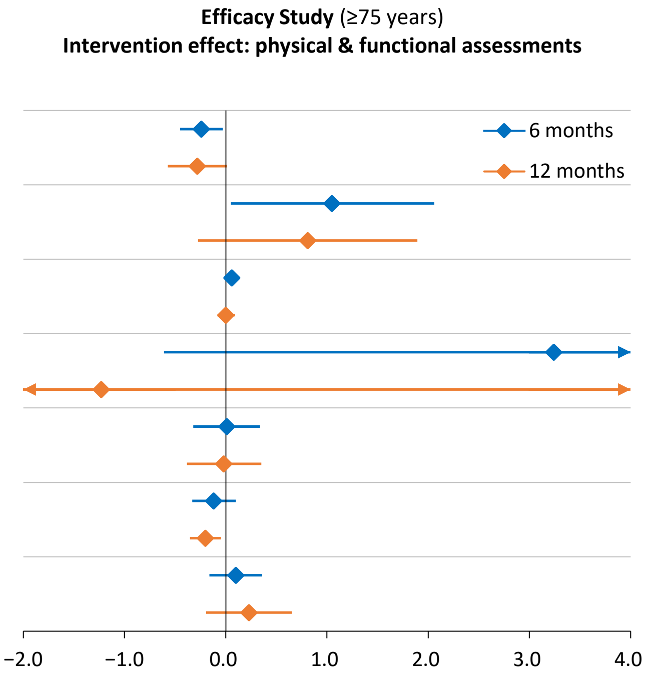

Metric	Assessment	Mean ± SD Control	Mean ± SD Intervention	Interaction (95% CI)[a]	*P*-value
CHS Frailty score	6 months	1.28 ± 1.13	1.07 ± 1.04	**−0.24 (−0.45, −0.03)**	**0.025**
	12 months	1.39 ± 1.13	1.06 ± 1.00	**−0.28 (−0.56, −0.00)**	**0.044**
Grip strength (kg)	6 months	21.55 ± 9.24	21.04 ± 6.86	**1.05 (0.05, 2.06)**	**0.039**
	12 months	21.08 ± 8.39	20.63 ± 7.03	0.81 (−0.26, 1.88)	0.139
Gait speed (m/s)	6 months	0.87 ± 0.30	0.96 ± 0.31	0.06 (−0.02, 0.13)	0.130
	12 months	0.89 ± 0.30	0.97 ± 0.31	0.00 (−0.07, 0.08)	0.915
Physical activity (MET)	6 months	12.9 ± 13.0	15.2 ± 19.7	3.24 (−0.61, 7.09)	0.099
	12 months	15.2 ± 24.0	14.4 ± 14.4	−1.23 (−7.72, 5.25)	0.707
Instrumental Activities of Daily Living	6 months	6.69 ± 1.67	7.08 ± 1.39	0.01 (−0.32, 0.34)	0.947
	12 months	6.82 ± 1.61	7.23 ± 1.30	−0.02 (−0.37, 0.34)	0.925
Geriatric Depression Scale-5	6 months	0.44 ± 0.90	0.40 ± 0.79	−0.12 (−0.33, 0.10)	0.279
	12 months	0.44 ± 0.93	0.35 ± 0.82	**−0.20 (−0.34, −0.06)**	**0.004**
Mini Nutritional Assessment Short Form	6 months	13.01 ± 1.41	13.04 ± 1.52	0.10 (−0.16, 0.36)	0.447
	12 months	12.90 ± 1.57	13.10 ± 1.43	0.23 (−0.18, 0.64)	0.272

[a]Linear mixed model adjusted for intervention, time and intervention/time interaction

Figure S3B Empowerment Study intervention effects on physical and functional performance among participants ≥75 years old

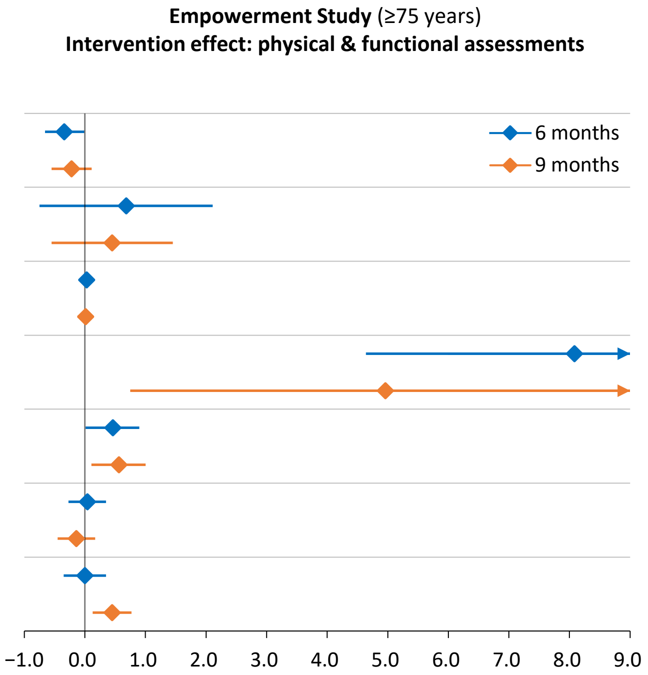

Metric	Assessment	Mean ± SD Control	Mean ± SD Intervention	Interaction (95% CI)[a]	*P*-value
CHS Frailty Score	6 months	1.27 ± 1.06	0.85 ± 0.94	**−0.34 (−0.66, −0.01)**	**0.044**
	9 months	1.20 ± 1.16	0.86 ± 0.99	−0.22 (−0.53, 0.09)	0.170
Grip strength (kg)	6 months	19.58 ± 6.69	20.62 ± 6.77	0.68 (−0.75, 2.11)	0.351
	9 months	19.84 ± 6.84	20.83 ± 6.36	0.45 (−0.53, 1.43)	0.364
Gait speed (m/s)	6 months	0.92 ± 0.32	1.01 ± 0.31	0.03 (−0.03, 0.10)	0.348
	9 months	0.91 ± 0.32	1.00 ± 0.31	0.01 (−0.10, 0.11)	0.884
Physical activity (MET)	6 months	12.5 ± 14.8	18.5 ± 30.5	**8.08 (4.64, 11.51)**	**<0.001**
	9 months	12.9 ± 17.3	16.2 ± 24.1	**4.96 (0.77, 9.15)**	**0.020**
Instrumental Activities of Daily Living	6 months	6.78 ± 1.61	7.02 ± 1.52	**0.46 (0.01, 0.90)**	**0.045**
	9 months	6.66 ± 1.74	7.05 ± 1.43	**0.56 (0.13, 0.98)**	**0.010**
Geriatric Depression Scale-5	6 months	0.44 ± 0.94	0.46 ± 0.88	0.04 (−0.27, 0.35)	0.808
	9 months	0.61 ± 1.00	0.44 ± 0.96	−0.14(−0.43, 0.15)	0.332
Mini Nutritional Assessment Short Form	6 months	12.87 ± 1.54	12.95 ± 1.17	0.00 (−0.35, 0.35)	0.999
	9 months	12.79 ± 1.71	13.32 ± 0.98	**0.45 (0.15, 0.75)**	**0.004**

[a]Linear mixed model adjusted for intervention, time and intervention/time interaction

Chen LK, et al. Efficacy of multidomain interventions to improve physical frailty, depression and cognition: data from cluster-randomized controlled trials.
J Cachexia Sarcopenia Muscle 2020; DOI: 10.1002/jcsm.12534.
Prof. Liang-Kung Chen, Center for Geriatrics and Gerontology, Taipei Veterans General Hospital, 201, Sec. 2, Shih-Pai Rd., Taipei 11217, Taiwan. Email lkchen2@vghtpe.gov.tw
