# Supplementary material for: Efficacy of multidomain interventions to improve physical frailty, depression and cognition: data from cluster‐randomized controlled trials
Source: J Cachexia Sarcopenia Muscle. 2020 Mar 5;11(3):650–62. doi: 10.1002/jcsm.12534 (PMC7296266; doi:10.1002/jcsm.12534)
Supplement: Supplementary file 8 — Figure S4. Intervention effects on cognitive performance among participants ≥75 years old [file JCSM-11-650-s005.pdf]

Figure S4A Efficacy Study intervention effects on cognitive performance among participants ≥75 years old

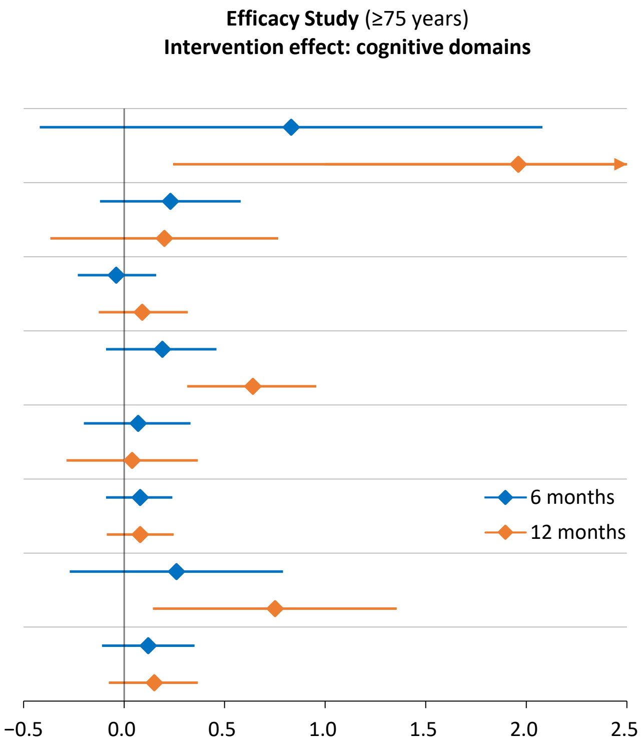

Metric	Assessment	Mean ± SD Control	Mean ± SD Intervention	Interaction (95% CI)[a]	*P*-value
Montreal Cognitive Assessment (adjusted)	6 months	17.71 ± 5.90	18.55 ± 6.05	0.83 (−0.42, 2.08)	0.183
	12 months	17.22 ± 6.36	19.92 ± 5.97	**1.96 (0.25, 3.68)**	**0.027**
Visuospatial executive	6 months	2.00 ± 1.68	2.48 ±1.61	0.23 (−0.12, 0.58)	0.195
	12 months	1.99 ± 1.68	2.63 ± 1.70	0.20 (−0.36, 0.76)	0.476
Naming	6 months	2.18 ± 1.01	2.13 ± 1.02	−0.04 (−0.23, 0.16)	0.713
	12 months	2.19 ± 1.06	2.37 ± 0.95	0.09 (−0.12, 0.31)	0.396
Concentration	6 months	4.13 ± 1.59	4.22 ± 1.64	0.19 (−0.09, 0.46)	0.182
	12 months	3.77 ± 1.82	4.45 ± 1.52	**0.64 (0.32, 0.95)**	**<0.001**
Language	6 months	1.21 ± 1.05	1.45 ± 1.01	0.07 (−0.20, 0.33)	0.623
	12 months	1.29 ± 1.06	1.51 ± 1.03	0.04 (−0.28, 0.36)	0.998
Abstract thinking	6 months	0.40 ± 0.67	0.49 ± 0.71	0.08 (−0.09, 0.24)	0.367
	12 months	0.35 ± 0.63	0.53 ± 0.78	0.08 (−0.08, 0.24)	0.332
Delayed recall	6 months	1.74 ± 1.79	1.88 ± 1.76	0.26 (−0.27, 0.79)	0.326
	12 months	1.63 ± 1.71	2.30 ± 1.89	**0.75 (0.15, 1.35)**	**0.015**
Orientation	6 months	5.09 ± 1.21	5.11 ± 1.15	0.12 (−0.11, 0.35)	0.316
	12 months	5.06 ± 1.15	5.20 ± 1.11	0.15 (−0.07, 0.36)	0.177

[a]Linear mixed model adjusted for intervention, time and intervention/time interaction

Figure S4B Empowerment Study intervention effects on cognitive performance among participants ≥75 years old

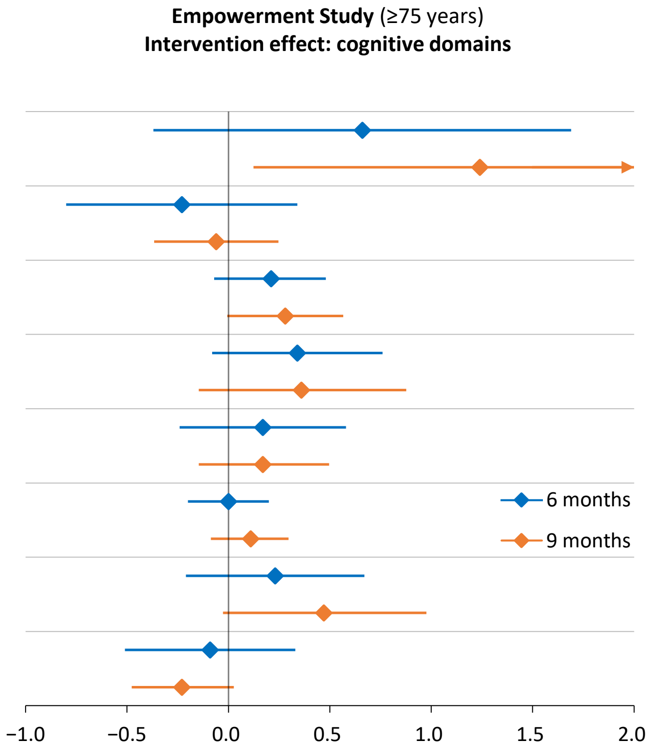

Metric	Assessment	Mean ± SD Control	Mean ± SD Intervention	Interaction (95% CI)[a]	*P*-value
Montreal Cognitive Assessment (adjusted)	6 months	18.86 ± 6.50	18.60 ± 6.02	0.66 (−0.37, 1.69)	0.209
	9 months	18.95 ± 6.35	20.05 ± 6.33	**1.24 (0.13, 2.35)**	**0.029**
Visuospatial executive	6 months	2.73 ± 1.54	2.31 ± 1.63	−0.23 (−0.80, 0.34)	0.424
	9 months	2.68 ± 1.49	2.62 ± 1.66	−0.06 (−0.36, 0.24)	0.694
Naming	6 months	1.90 ± 1.06	1.83 ± 1.10	0.21 (−0.07, 0.48)	0.130
	9 months	1.88 ± 1.03	2.06 ± 1.04	0.28 (0.00, 0.56)	0.054
Concentration	6 months	4.17 ± 1.78	3.95 ± 1.66	0.34 (−0.08, 0.76)	0.108
	9 months	4.08 ± 1.72	4.09 ± 1.84	0.36 (−0.14, 0.87)	0.152
Language	6 months	1.47 ± 0.92	1.59 ± 0.97	0.17 (−0.24, 0.58)	0.418
	9 months	1.61 ± 1.01	1.78 ± 0.92	0.17 (−0.14, 0.49)	0.286
Abstract thinking	6 months	0.48 ± 0.72	0.36 ± 0.64	0.00 (−0.20, 0.20)	0.971
	9 months	0.44 ± 0.75	0.52 ± 0.76	0.11 (−0.08, 0.29)	0.256
Delayed recall	6 months	2.11 ± 1.84	2.47 ± 1.66	0.23 (−0.21, 0.67)	0.306
	9 months	2.27 ± 1.76	2.94 ± 1.76	0.47 (−0.02, 0.97)	0.061
Orientation	6 months	5.03 ± 1.34	5.12 ± 1.07	−0.09 (−0.51, 0.33)	0.678
	9 months	5.03 ± 1.25	5.09 ± 1.26	−0.23 (−0.47, 0.02)	0.072

[a]Linear mixed model adjusted for intervention, time and intervention/time interaction

Chen LK, et al. Efficacy of multidomain interventions to improve physical frailty, depression and cognition: data from cluster-randomized controlled trials.
J Cachexia Sarcopenia Muscle 2020; DOI: 10.1002/jcsm.12534.
Prof. Liang-Kung Chen, Center for Geriatrics and Gerontology, Taipei Veterans General Hospital, 201, Sec. 2, Shih-Pai Rd., Taipei 11217, Taiwan. *Email* lkchen2@vghtpe.gov.tw
